# Inequality and cooperation in social networks

**DOI:** 10.1038/s41598-022-10733-8

**Published:** 2022-04-26

**Authors:** David Melamed, Brent Simpson, Bradley Montgomery, Vedang Patel

**Affiliations:** 1 Department of Sociology, The Ohio State University, Columbus, OH 43210 USA; 2 Core Faculty, Translational Data Analytics Institute, The Ohio State University, Columbus, OH 43210 USA; 3 Department of Sociology, University of South Carolina, Columbia, SC 29208 USA; 4 Microsoft, Redmond, WA 98052 USA

**Keywords:** Human behaviour, Social evolution

## Abstract

Social networks are fundamental to the broad scale cooperation observed in human populations. But by structuring the flow of benefits from cooperation, networks also create and sustain macro-level inequalities. Here we ask how two aspects of inequality shape the evolution of cooperation in dynamic social networks. Results from a crowdsourced experiment (N = 1080) show that inequality alters the distribution of cooperation within networks such that participants engage in more costly cooperation with their wealthier partners in order to maintain more valuable connections to them. Inequality also influences network dynamics, increasing the tendency for participants to seek wealthier partners, resulting in structural network change. These processes aggregate to alter network structures and produce greater system-level inequality. The findings thus shed critical light on how networks serve as both boon and barrier to macro-level human flourishing.

## Introduction

Both cooperation^1–5^ and resource inequality^6,7^ are universal but variable across human societies. Although several studies have suggested that variation in material wealth inequality and cooperation may be linked^2,8–10^, we currently know little about how inequality in material wealth or endowments impacts cooperation^8^ or how cooperation, in turn, influences inequalities^11^. We study the bidirectional effects of cooperation and inequality in dynamic networks, where ties between alters represent opportunities to cooperate^8,12–14^.

Two interrelated bases of inequality are likely fundamental for cooperation in social networks. First, inequality in *wealth* may lead to differences in self-reliance, which decreases the tendency for the wealthy to cooperate or form ties to new partners, especially with the poor. Some evidence supports the contention that visible inequality results in the wealthy being less cooperative^8,9^. Alternatively, wealth may lead to noblesse oblige, leading the rich to be more generous in their interactions with their less fortunate network partners. There is theory and evidence supporting this view as well^10^. We refer to any tendency for greater wealth to affect one’s behavior as *baseline wealth effects*.

We argue that wealth will affect both cooperation and network dynamics beyond its effects on the holder of wealth. Here we test the argument that the tendency for people to derive greater benefits from interactions with wealthy partners^10,15^ can generate differences in cooperation, altering network dynamics and exacerbating existing inequalities in networks. That is, a key starting point for our investigation is that cooperators who possess more material wealth (or other valuable resources like technology or knowledge) can generate larger material benefits for their network partners than cooperators with access to less material wealth. Thus, collaborations with individuals with more wealth or other valuable resources (e.g., greater human capital) generally result in higher overall outcomes. For example, one will likely benefit more from investing in a business venture with a wealthy partner than a poor partner, or by working on a project with an experienced vs. inexperienced collaborator. Following related work, we refer to this second aspect of inequality as *wealth productivity effects*^10^.

Critically, however, interactions with the wealthy are only more productive if the wealthy are cooperative. One does not benefit from collaborating with a wealthy or experienced partner who freerides on one’s efforts while contributing nothing of their own. We expect that wealth productivity effects will lead to more cooperation with the wealthy, both to maintain ties to them and to bring about higher levels of cooperation from them. Thus, when we account for wealth productivity effects, we expect that these higher levels of preferential attachment to the rich and cooperation with them will lead to “rich-get-richer effects.” This, in turn, will result in increased network-level inequality. This is consistent with recent work^16^ showing that the greater resources of the wealthy allow them to produce larger benefits for interaction partners for any given level of cooperation. Those interaction partners, in turn, attribute higher levels of cooperativeness to the wealthy than their (equally cooperative) poorer counterparts, leading the wealthy to gain more reputational benefits, which can then lead to increased monetary rewards in downstream interactions.

Summing up, we study not only the effects of wealth inequality and productivity on cooperation in dynamic networks, but also how those factors lead to concentration of both material wealth and social wealth (i.e., social ties). More specifically, we conducted a large-scale behavioral experiment using human subjects to answer several interrelated questions about how inequality shapes cooperation, network dynamics, and macro-level inequalities: (1) How do baseline wealth and wealth productivity affect cooperation in networks? (2) Does wealth productivity increase cooperation because participants give more to wealthy partners? (3) Does wealth productivity lead to preferential attachment to the rich? If so, does inequality in network degree increase? And, (4) Do the effects of inequality on cooperation and network dynamics combine to increase network-level inequality? That is, does wealth productivity lead to “rich get richer” effects by shaping who cooperates with whom?

A total of 1080 participants were embedded in 40 dynamic networks (average initial network size = 27; “Methods”). Initial networks were random (Erdös-Rényi) graphs, with a density of 0.167 or about 4 ties each. Each network tie represented an opportunity to interact in an iterated prisoner’s dilemma (PD). In each round, each participant made a single decision to give 0 to 50 monetary units (MUs; in ten-unit increments) to all of their alters^8,12,13^, where 0 represented full defection and 50 represented maximal cooperation. Specifically, consistent with a PD incentive structure, any given person in an interaction benefited most when they gave nothing and the other gave maximally (the "T" payoff); joint benefit was highest when both gave maximally (the "R" payoff); joint benefit was lowest when both gave nothing (the "P" payoff); and the worst individual outcome alter gave nothing and ego gave maximally and (the "S" payoff). As a PD, T > R > P > S. After each round, participants were told how much each of their partners had cooperated and how much they benefited from each partner’s cooperation.

Participants could sever one tie and propose a new one every three rounds. Ties could be severed unilaterally, but proposed new ties required approval by the selected other. Participants were not informed that tie updates occurred regularly. When given the opportunity to add ties, participants saw alters’ participant ID, their endowment, and how much that alters’ partners had received from them in the previous round (Supplementary Materials). The study lasted 19 rounds. But to avoid end game effects, participants were not told this.

Our experiment was a 2 × 2 design. We manipulated whether there was baseline wealth inequality in the initial endowment distribution and whether network relations were characterized by wealth productivity. In the baseline wealth equality condition, all participants received 1000 MUs at the beginning of the study^12–14^. In the baseline wealth inequality condition, the endowment distribution had an average of 1000 MUs, but a Gini coefficient of 0.3, comparable to Austria, Poland and Hungary^17^ and substantially less than the U.S. (Gini = 0.41). Endowments were randomly assigned to participants. Participants could see the endowments of those to whom they were connected. They could also see the endowments of non-partners in the tie selection phase. Thus, they had a rough sense of the distribution of endowments.

**Figure 1:**
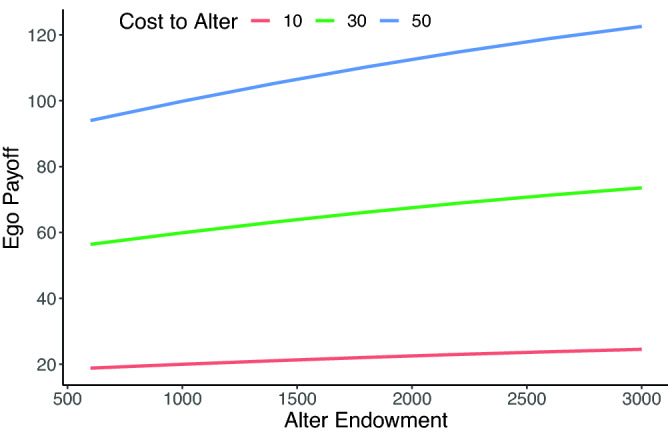
Illustration of our wealth productivity manipulation. As alters endowment increases, so does the amount ego receives from varying levels of alter’s cooperation (10, 30, and 50).

Our key manipulation, wealth productivity, was whether the effect of alter’s cooperation on ego’s outcome was a function of alters’ endowment. As is standard in the literature^12–14^, in the control condition, each MU given to alter was doubled. In the wealth productivity treatment condition, however, the amount each alter received from ego’s cooperation was a function of ego’s endowment: the larger ego’s endowment, the more the alter benefited from ego’s cooperation. Figure 1 illustrates how ego’s endowment and cooperation level shape partner outcomes. Conceptually the wealth productivity condition captures a basic feature of much of social life: interacting with wealthier partners (or partners who are higher in human capital) can bring larger benefits than interacting with less wealthy partners, but only if those wealthy partners are cooperative. As in real world interactions, the wealth productivity manipulation infuses value (endowments) into the social system when wealthy actors are cooperative. But, as a conservative test of our arguments, we adjusted the endowments to remove these effects when investigating network-level inequality (Supplementary Materials).

## Results

### Cooperation

**Figure 2:**
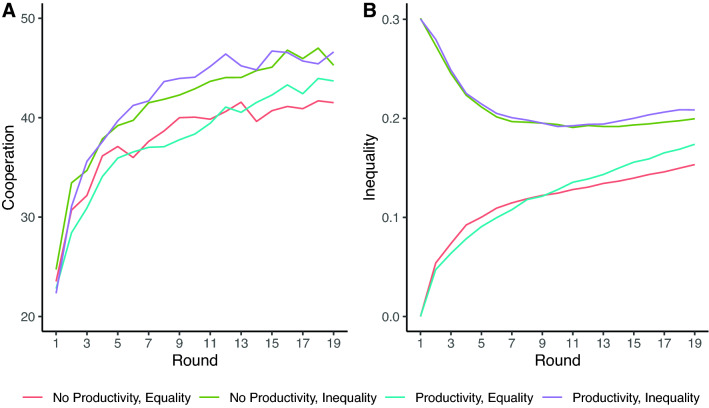
Average (**A**) cooperation and (**B**) inequality (Gini coefficient) by experimental condition through time. *No Productivity, Equality* indicates no wealth productivity and baseline endowment equality. *Productivity, Inequality* indicates wealth productivity and baseline endowment inequality.

Figure 2A shows average rates of cooperation through time by experimental condition. Consistent with past work on dynamic networks, we find that cooperation increases quickly^12–14^ and that initial endowment inequality induces additional cooperation^8,9^. Wealth productivity enhances cooperation early on when there is endowment inequality, and increases cooperation in later rounds when there is initial endowment equality, a pattern we describe in more detail below.

Table 1 presents the results from four models of cooperation at three different levels of analysis. In Model 1, we examine how our experimental manipulations are related to average rates of cooperation over the last four rounds, i.e., after cooperation rates have stabilized. Consistent with some past work^8^ we find that initial baseline inequality promotes cooperation in our social systems. While the wealth productivity manipulation increases cooperation, it is not significant; as we report below, however, this is due to ceiling effects on cooperation specifically in these latter rounds of our study.

**Table 1:** Summary of regression models predicting cooperation at 3 different levels of analysis.

	Model 1	Model 2	Model 3	Model 4
Wealth productivity (W)	1.002 (1.606)	− 5.146* (2.370)	− 9.496*** (2.986)	0.169 (1.624)
Baseline inequality (B)	3.282* (1.606)	1.365 (2.370)	3.300 (3.712)	3.501** (1.624)
Round (R)		0.248*** (0.051)	0.367*** (0.032)	0.233*** (0.023)
Gini coefficient (G)			− 24.203 (13.320)	
Alter endowment (A)				− 1.433*** (0.385)
W × B		7.260* (3.352)	13.098** (5.407)	
W × R		0.399*** (0.072)		
B × R		0.145* (0.072)		
W × B × R		− 0.485*** (0.102)		
W × G			68.169*** (14.722)	
B × G			7.102 (18.223)	
W × B × G			− 80.240** (24.745)	
W × A				1.110* (0.446)
B × A				1.099* (0.446)
Intercept	42.299*** (1.391)	37.679*** (1.676)	39.375*** (2.259)	37.042*** (1.438)
Level 2 variance component		23.33	23.85	22.32
Level 3 variance component				72.06

N Model 1 = 40 networks, N Models 2 and 3 = 520 network-rounds. N Model 4 = 10,185 network-participant-rounds. *p < 0.05, **p < 0.01, ***p < 0.001.

**Figure 3:**
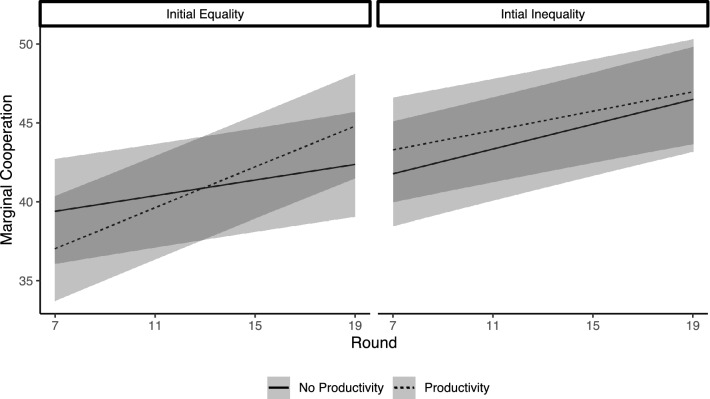
Marginal cooperation illustrating the interaction between wealth productivity, baseline inequality and round.

Next we look at how cooperation unfolds in networks over time. Here we find that both of our inequality manipulations interact with time to shape cooperation trajectories. Model 2 of Table 1 presents these parameter estimates and Fig. 3 illustrates the implications of the interaction effects. In networks with an initially equal baseline endowment distribution, we observe that wealth productivity has a positive effect only once inequality emerges in later rounds. On the other hand, in networks with an initially unequal baseline endowment distribution we observe that wealth productivity has a positive effect early on that is diminished later in the study. This is likely due to a ceiling effect, given the very high rates of cooperation in later rounds. This diminishment at the end of the study is why we do not observe a main effect of wealth productivity at the network level of analysis (i.e., Model 1).

**Figure 4:**
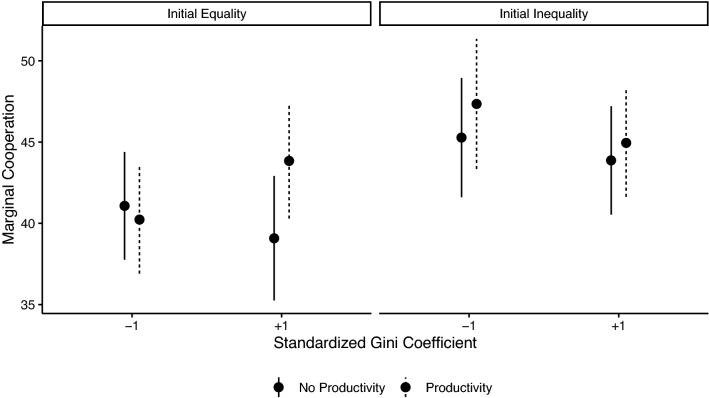
Marginal means from Model 3 of Table 1 illustrating the interaction between wealth productivity, baseline (manipulated) inequality, and observed network-level inequality.

Building on the insights in Fig. 3, we find that network-level inequality, as measured by the Gini coefficient, moderates the interaction between our two experimental manipulations. Further, this interaction is not moderated by round (Supplementary Materials). Model 3 in Table 1 presents a summary of the parameter estimates and Fig. 4 presents marginal means from the model to illustrate the interaction effect. We find that wealth productivity promotes cooperation after the emergence of inequality in initially equal systems. And in part due to the ceiling effect described above, wealth productivity only has a small positive effect on cooperation in initially unequal systems. That is, our data suggests that the presence of wealth productivity in human social relations promotes cooperation when there is inequality in the system (and when network dynamics have not induced a cooperation ceiling).

**Figure 5:**
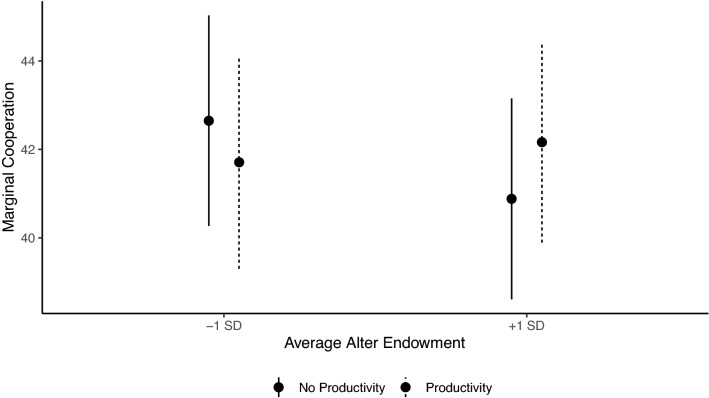
Marginal means from Model 4 of Table 1 illustrating the interaction between wealth productivity and average alter endowment.

In terms of why cooperation is higher in the wealth productivity condition, we predicted participants would give more to wealthier partners in the presence of wealth productivity effects. This is because investing more in “higher value” relations follows from a greater incentive to maintain ties with those relations that have the potential to benefit them more^18,19^. As illustrated in Fig. 5, regardless of whether the networks entailed initial baseline endowment inequality, we find that participants in the control condition give less to their wealthier alters, whereas participants in the wealth productivity condition gave more to their wealthy alters. Relative to the control, wealth productivity increases cooperation when alters are relatively rich (Table 1, Model 4, b = 1.11, se = 0.45, *p* = 0.02).

### Network dynamics

**Figure 6:**
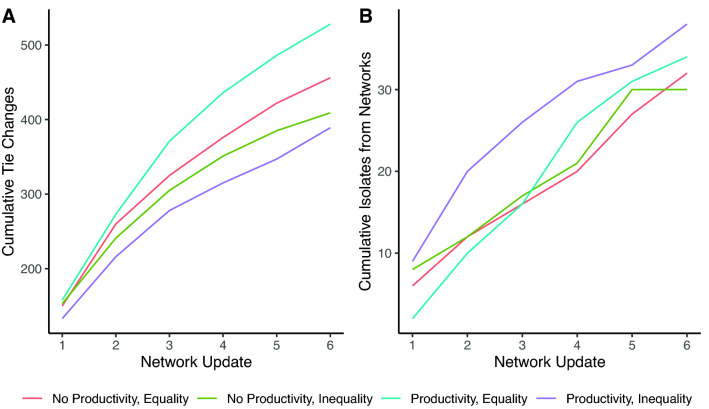
Cumulative (**A**) tie updates and (**B**) network isolates by experimental condition through time.

Figure 6 shows descriptive statistics for network dynamics. Most network change happened in earlier rounds, as indicated by fewer decisions to drop a partner and select a new one in later rounds (see slope declines in Fig. 6A). In terms of network exclusion (Fig. 6B), participants appear to be consistently at risk throughout the study, with heightened risk in the wealth productivity and endowment inequality condition (purple line). Below we report results of statistical models adjusting for participant behaviors on condition-level effects on network dynamics that confirm this.

When altering their ties, participants decided whether to drop an alter (Fig. 6A). If they elected to drop an alter, they then indicated which alter to drop. Participants could then decide which new alter to add. If the proposed tie was confirmed by the alter, a new tie was formed. (That is, severing ties was a unilateral decision, but forming new ties was bilateral). Aside from these individual decisions, this process also resulted in some participants being excluded from the entire network (Fig. 6B). After adjusting for the effect of time and cooperation (Supplementary Material), we find that participants in the baseline endowment inequality condition were less likely to drop alters (Table S1, Model 2: b = − 0.35, se = 0.11, *p* = 0.002), but that those in the wealth productivity condition did not differ from the control. Conditional on dropping a partner, net of cooperativeness, participants were more likely to drop wealthier alters. While this effect is diminished in the wealth productivity condition, the decrease is not statistically significant (Table S2, Model 2: b = − 0.069, se = 0.069, *p* = 0.32).

**Figure 7:**
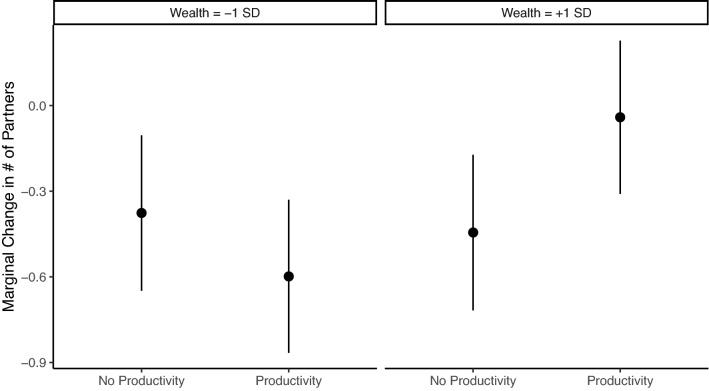
Marginal change in number of partners illustrating the interaction between participant baseline wealth and wealth productivity.

Wealth productivity has a strong effect on new partner selection, however. While participants selected wealthier alters in the control condition (Table S3, Model2: b = 0.288, se = 0.036, *p* < 0.001), the effect of alter wealth was more than twice as strong in the wealth productivity condition (b = 0.295, se = 0.050, *p* < 0.001). Thus, while decisions to sever a tie were primarily driven by alter’s cooperation, the formation of new partners was based on the prospective alter’s cooperativeness and wealth. Most importantly, wealth particularly mattered when it had an impact on ego’s welfare, i.e., in the wealth productivity condition. To illustrate, Fig. 7 shows the marginal change in number of partners as a function of participant wealth and whether they were in the wealth productivity condition. Most change is negative since networks got smaller, on average, over time. We find no difference by condition in the decrease in number of partners for relatively poor participants, but wealthier participants in the wealth productivity condition lose significantly fewer partners on average than wealthier participants in the control condition. This preferential attachment to rich alters resulted in structural change to the networks, with networks in the wealth productivity condition acquiring higher inequality in the degree distribution than those in control networks (Fig. S7). This means that not only was wealth increasingly concentrated; relationships were increasingly concentrated as well.

**Figure 8:**
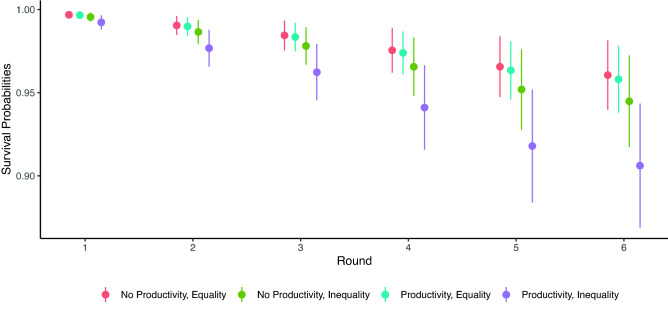
Cox Proportional Hazard model survival probabilities at each network update by experimental condition. Participant endowments and cooperation (time-varying covariates) are set to their means.

Above we focused on the number of ties among active members of our networks. Another important consequence of these network dynamics is that 15.5% of participants became isolated from their network. These participants were therefore excluded from the analyses above. Here, we focus on the hazard of having no ties, i.e., becoming excluded. We find that despite cooperating at higher rates (Fig. S9), participants in the combined wealth productivity and baseline inequality condition were significantly less likely to complete all rounds before becoming isolated. Figure 8 presents survival probabilities by experimental condition for each tie update opportunity from a Cox proportional hazards model (Supplementary Material). As illustrated, after adjusting for the fact that those in the wealth productivity/baseline inequality condition cooperated at higher rates than other isolates, they are still at increased risk of network exclusion.

### Inequality

Figure 2B shows average inequality in endowments, as measured by the Gini coefficient, through time by experimental condition. Consistent with closely related work^8^, the networks we study produce a constrained range of inequality. As detailed in the Methods, we ran a numerical simulation to determine how much inequality is plausible based on our experimental setting. We assumed networks of the same size and density as used in our experiment, with very similar endowment distributions, and the same constraint on agent behavior to cooperate in 10-point increments (see Supplementary Materials for full details on the simulation). We find that 95% of the resulting Gini coefficients fall within the range of 0.07 to 0.27. With this as reference for understanding inequality in these social systems, we turn to factors shaping observed inequality in our experiments with human participants.

**Figure 9:**
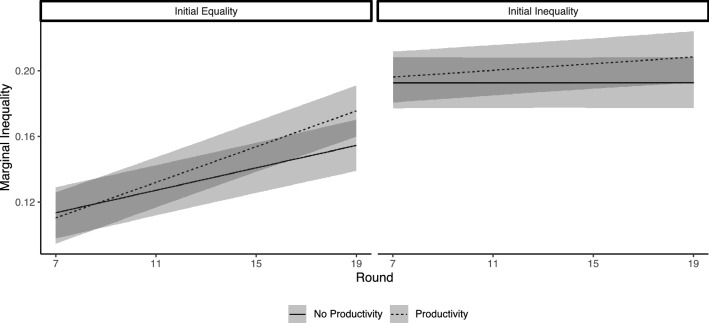
Marginal Inequality depicting the interaction between round, initial baseline endowment inequality, and wealth productivity.

We find, unsurprisingly, that initial baseline endowment inequality is strongly related to network-level inequality at the end of our study (Table S8, Model 1: b = 0.044, se = 0.009, *p* < 0.001). More importantly, networks in the wealth productivity condition have increased inequality at the end of the study (b = 0.016, se = 0.009, *p* = 0.08). To illustrate how these patterns emerged, we modeled network-level inequality through time. We find that time interacts with both manipulated factors in our experiment. Figure 9 illustrates the patterns, showing how initial baseline endowment inequality shapes the trajectories, and that wealth productivity increases inequality through time.

## Discussion

We bring a fundamental feature of human interaction to bear on models of networks and cooperation: Interactions with the wealthy can generate far greater benefits to interaction partners than interactions with the disadvantaged, but only if those collaborators are cooperative. We independently manipulated two dimensions of wealth to allow us to assess how each impacted cooperation, network dynamics and inequality in networks. First, following^8–10^, we manipulated baseline wealth inequality, specifically whether some participants had higher endowments than others. We also manipulated wealth productivity^10^, which allowed us to model the tendency for people to benefit more from cooperative interactions with those who have greater material wealth or other valuable resources (e.g., greater human capital).

Our results show that these relational inequalities pattern cooperation, network dynamics, and network inequalities in important ways. Consistent with previous work, we find that initial endowment inequality promotes cooperation. We also find that inequality in wealth productivity promotes cooperation when there is inequality in the system. This is because participants cooperate at higher levels with the “rich” than the “poor.” These relational inequalities also shape network dynamics. Existing work shows that participants select on wealthier alters. Our results show that these effects are more than doubled when we allow for wealth productivity effects. What is more, this process resulted in meso-level structural change in the wealth productivity condition, with the networks increasing in degree inequality. That is, wealth productivity effects altered the topology of dynamic networks such that wealthier participants had more partners, even in the brief timeframe of our experiment. Finally, we find that initial endowment inequality impacts inequality at the conclusion of our study, and that wealth productivity inequalities contributes marginally to network-level inequality.

One simplifying assumption of our work is that participant make a single decision to cooperate that applies to all alters. While this is consistent with much of the past work on networks and cooperation^12,13^, allowing participants to make individualized decisions with each of their alters would have enabled them them to engage in conditional cooperation^14^. That is, participants could not directly reciprocate any given alter’s actions because they made a signle decision vis-à-vis all those to whom they were connected. We expect that this *reduced* wealth productivity effects in our study, because participants could not exclusively give more to the wealthy in their networks. But ultimately this is empirical question that can—and should—be addressed in future work.

Our findings suggest important caveats to current thinking on networks and cooperation. While social networks clearly promote cooperation, accounting for inequalities in wealth and wealth productivity leads to predictable asymmetries in cooperation. These asymmetries lead the resource rich to experience more benefits, including both material wealth and social ties. The resource poor, on the other hand, are more apt to be pushed to the peripheries of networks and even excluded altogether, despite their higher levels of cooperation. Accounting for the ubiquitous tendency toward inequality in human populations thus reveals a double edge of social networks for human cooperation. It also shows how the cooperation enhancing effects of social networks can lead to cumulative advantage for the wealthy^2,20–22^ and to sustained inequalities.

## Methods

### Data procurement

The Institutional Review at the University of South Carolina reviewed and approved this research. There was no deception. The experiment was conducted using Prolific, an online crowd-sourcing platform that has responded to concerns about data quality on other crowdsourcing platforms; for example, Prolific monitors users for unusual patterns, accounts are verified in multiple ways, the number of accounts per IP address are restricted, and payment accounts must be unique for each participant. Participants followed a link from Prolific to our custom Web app (see Supplementary Materials for screenshots of the app). They first completed a consent form, detailing study procedures, the approximate length of the study, and their expected payment. If they agreed to participant, they read instructions, completed comprehension check questions, and then the app randomly embedded them in a social network and tracked their behaviors over the course of study. Data were collected during the summer of 2020. Each session lasted ~ 40 min. Participants were paid $2.00 for completing the instructions and getting at least four of five comprehension check items correct, plus a bonus based on how many Monetary Units (MUs) they acquired throughout the study (1000 MUs = $1.00).

### Statistical analysis

We relied on linear statistical models for much of our analysis. We modeled cooperation as a continuous outcome. At the network-level of analysis, we modeled rates of cooperation, averaged over the last four rounds, as a function of experimental manipulations using OLS regression (N = 40). At lower levels of analysis, i.e., time in networks, or time in people in networks, we used random intercept mixed effects regression models, with the corresponding estimated variance components reported in the Tables. The repeated binary decision of whether to drop an alter was modeled using mixed effects logistic regression, with a random intercept for decisions nested within participants. Choices of which particular alter to drop, and then which new alter to select were modeled with fixed effects or conditional logistic regression. As detailed in the Supplementary Materials, network isolation was modeled using Cox proportional hazards models. Participants were at risk of isolation across each of the six possible network updates. We estimate the hazard of becoming isolated from the network as a function of experimental manipulations, and time-varying covariates for the participant’s endowment and cooperativeness over the last three rounds. In terms of network-level inequality, we first examined average inequality over the last four rounds using OLS regression (N = 40 networks). We then examined trends in inequality through time. To do so we modeled time in networks with random intercept mixed effects regression models. The figures illustrating the patterns in our models rely on estimated marginal means or probabilities; estimates of uncertainty are 95% confidence intervals estimated using the Delta method (Supplementary Materials).

## Supplementary Information

Supplementary Information.
