# Supplementary material for: Inequality and cooperation in social networks
Source: Sci Rep. 2022 Apr 26;12:6789. doi: 10.1038/s41598-022-10733-8 (PMC9042846; doi:10.1038/s41598-022-10733-8)
Supplement: Supplementary file 1 — Supplementary Information. [file 41598_2022_10733_MOESM1_ESM.docx]

Supplementary Materials for

**Inequality and Cooperation in Social Networks**

David Melamed,^*^ Brent Simpson,^*^ Bradley Montgomery, and Vedang Patel

*Email [melamed.9@osu.edu](mailto:melamed.9@osu.edu) & [bts@mailbox.edu](mailto:bts@mailbox.edu)

**Supplementary Text**

We adapted a version of Breadboard (McKnight and Christakis 2016) to include our wealth productivity manipulation. Below we show screenshots from our version of the software, illustrating the instructions and the interactional component of the study.

As illustrated below, when participants were deciding whether/how much to cooperate with their alters they were shown their alters endowments, and when relevant, how much alter gave ego on the last round. When selecting new partners, participants were shown the endowments and how much alters received from them on the previous round, but participants were given no information about the network structure.

*Study Instructions*


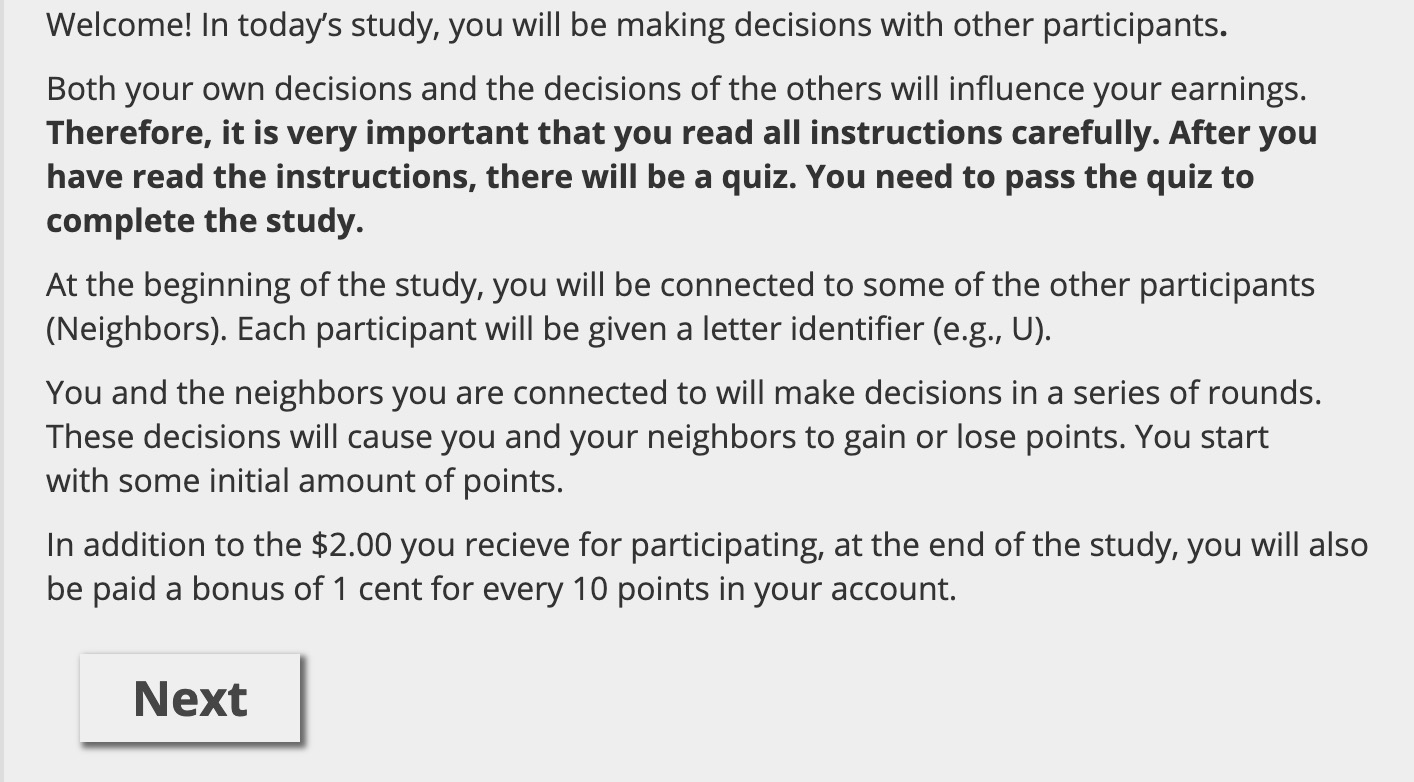


*Endowment Inequality Condition Instructions*


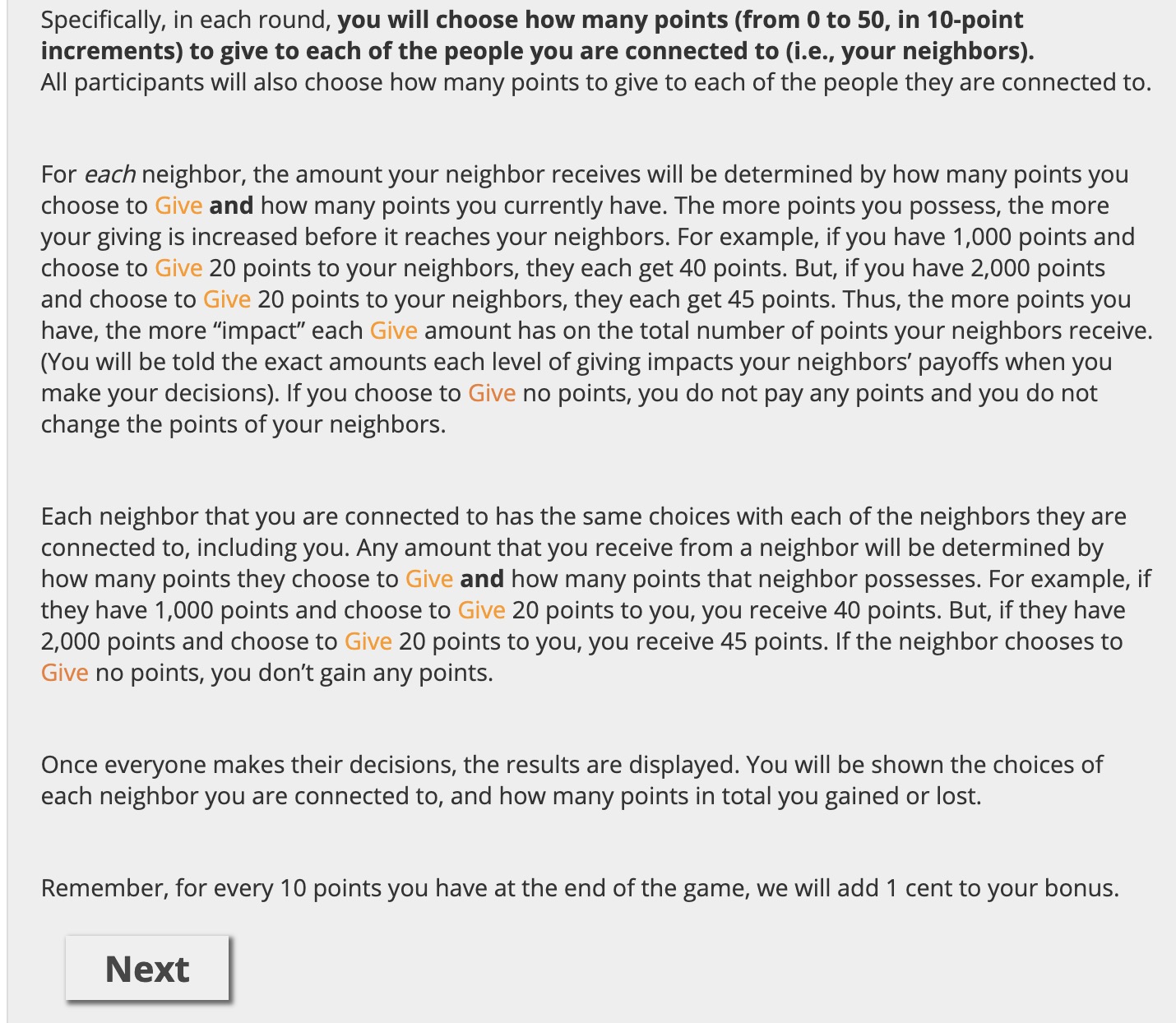


*No Endowment Inequality Condition Instructions*


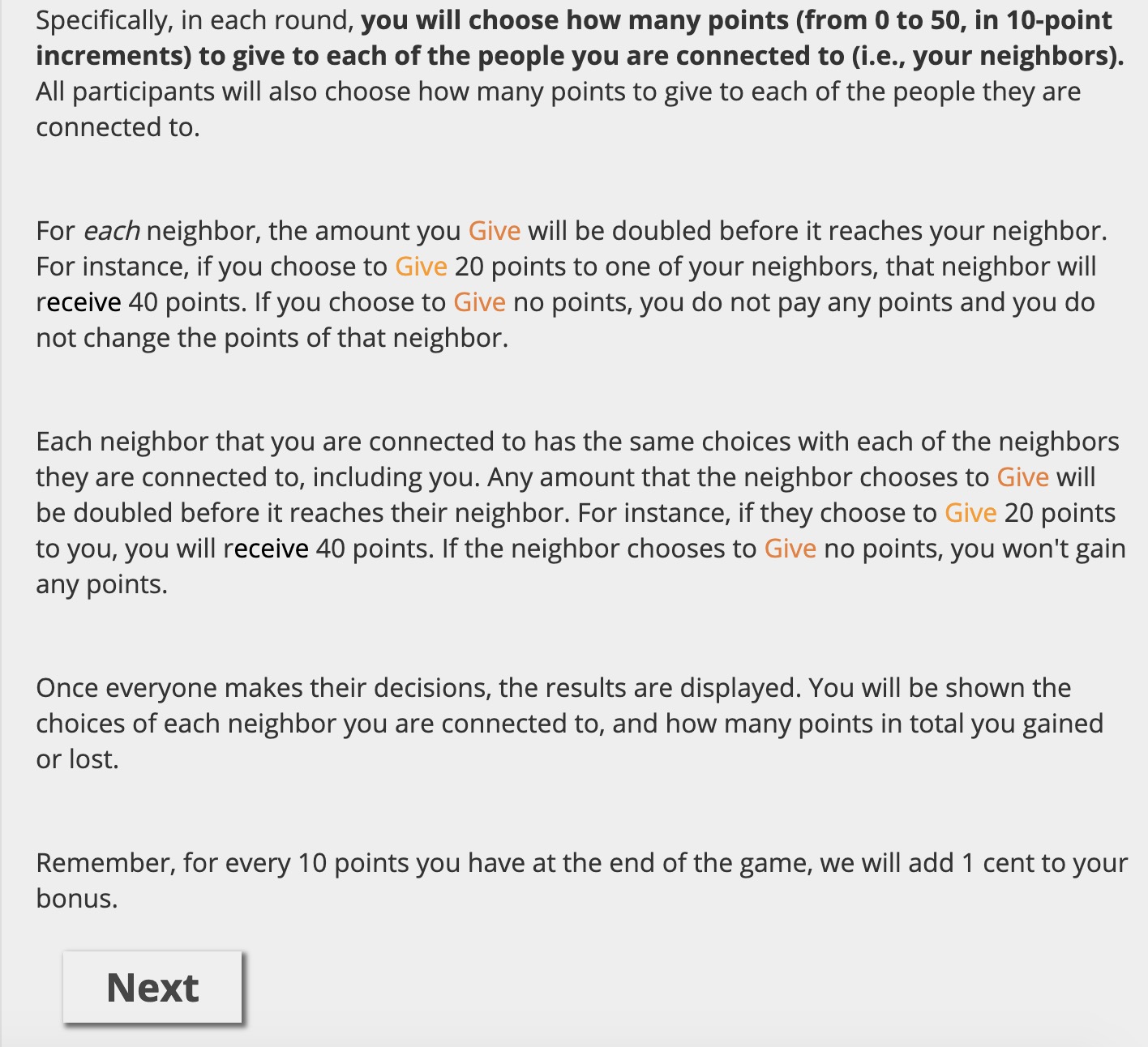


*Instructions on Network Dynamics*


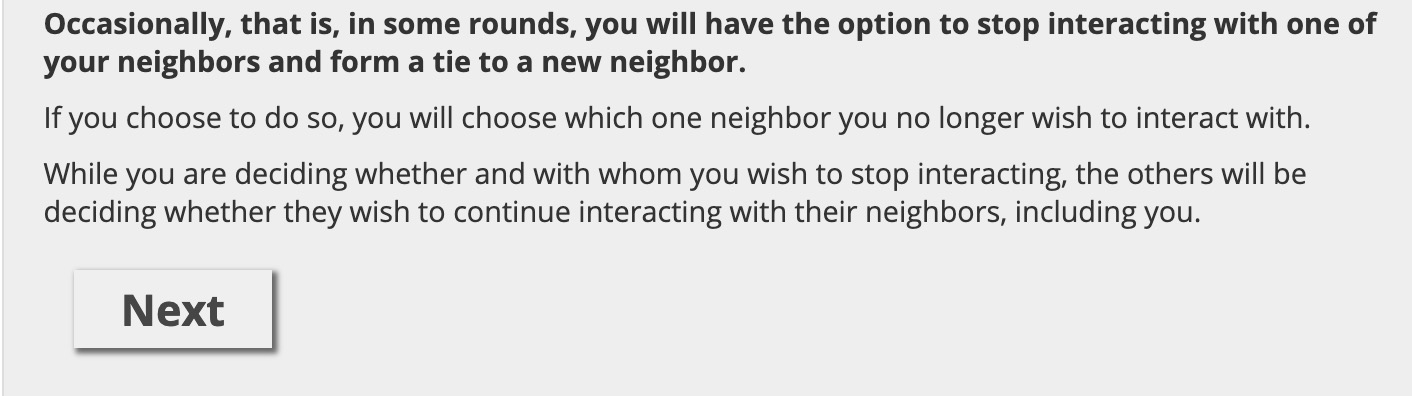


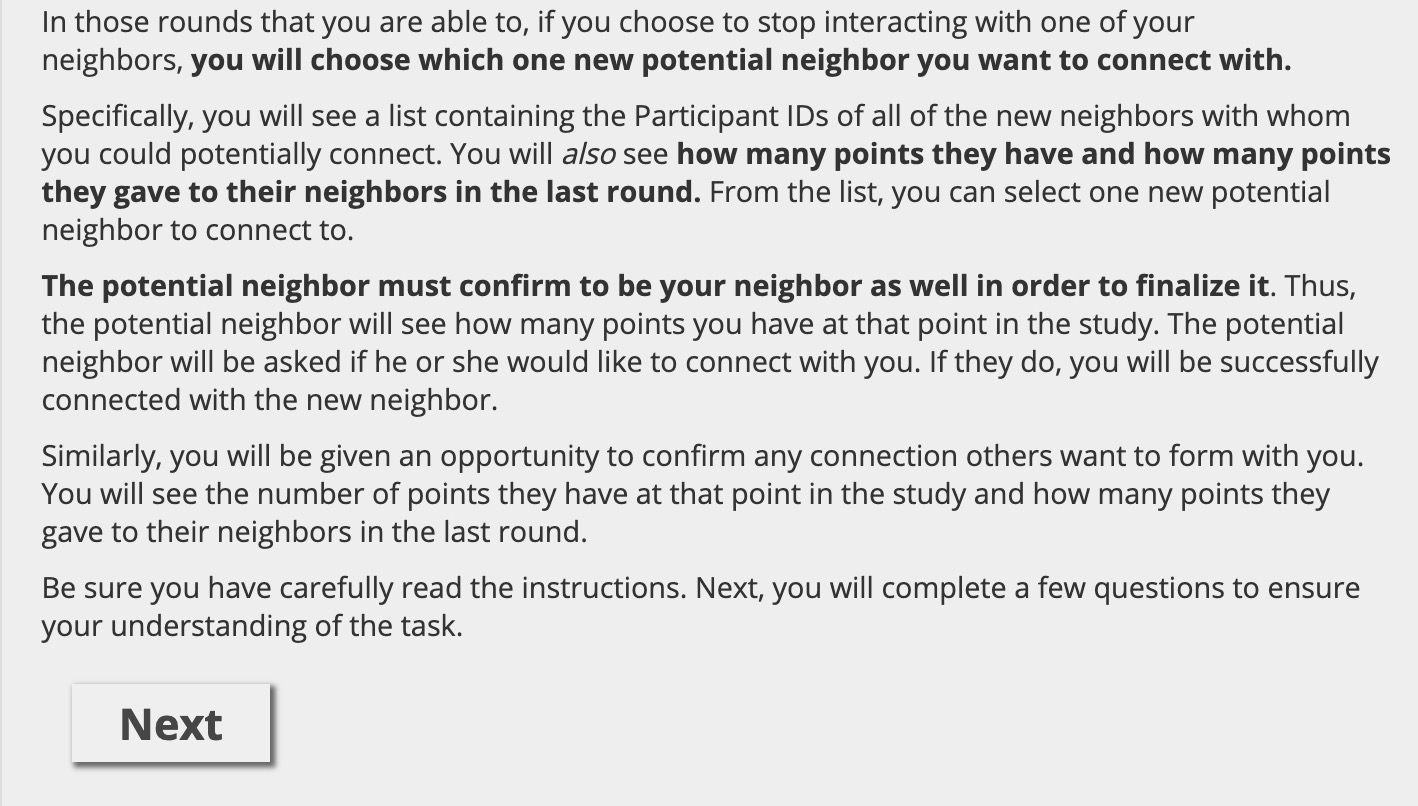


*Comprehension Check*


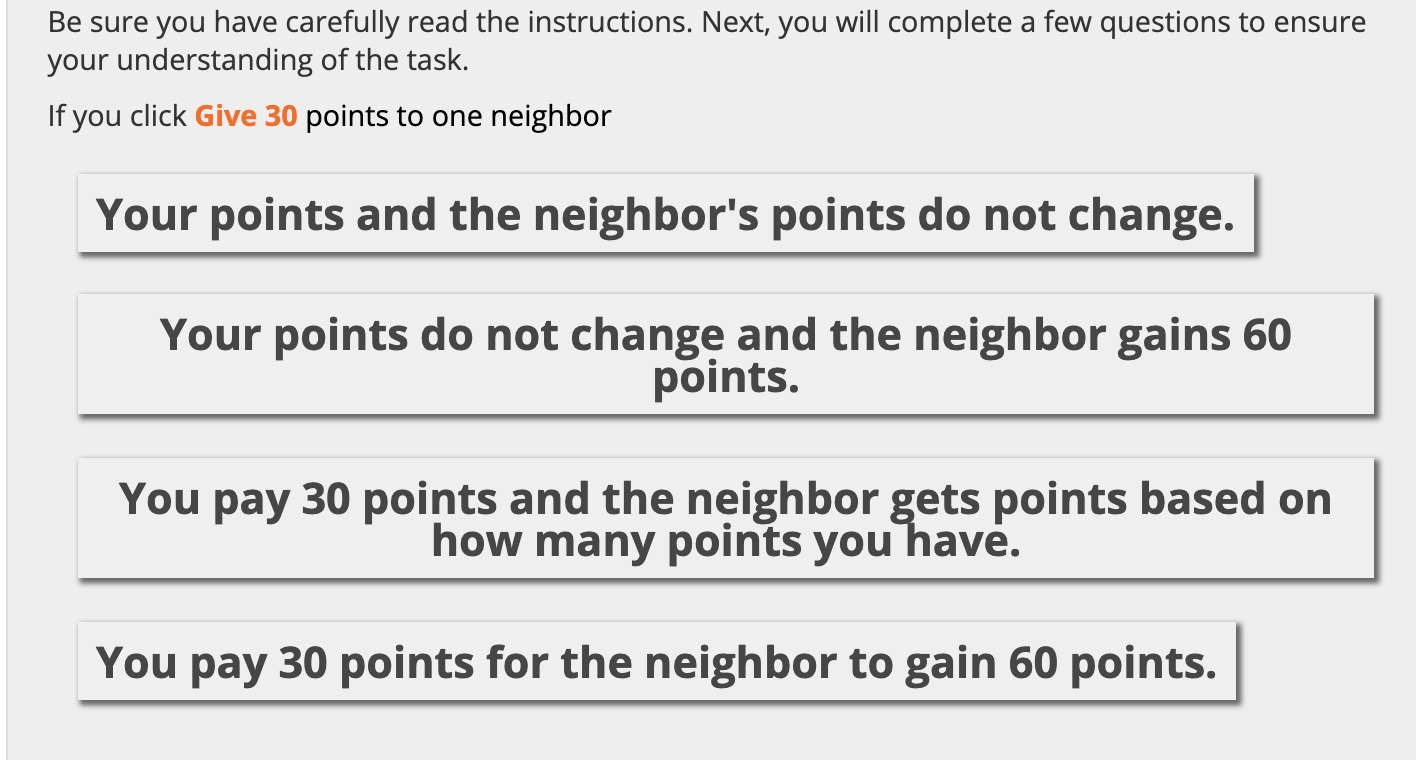


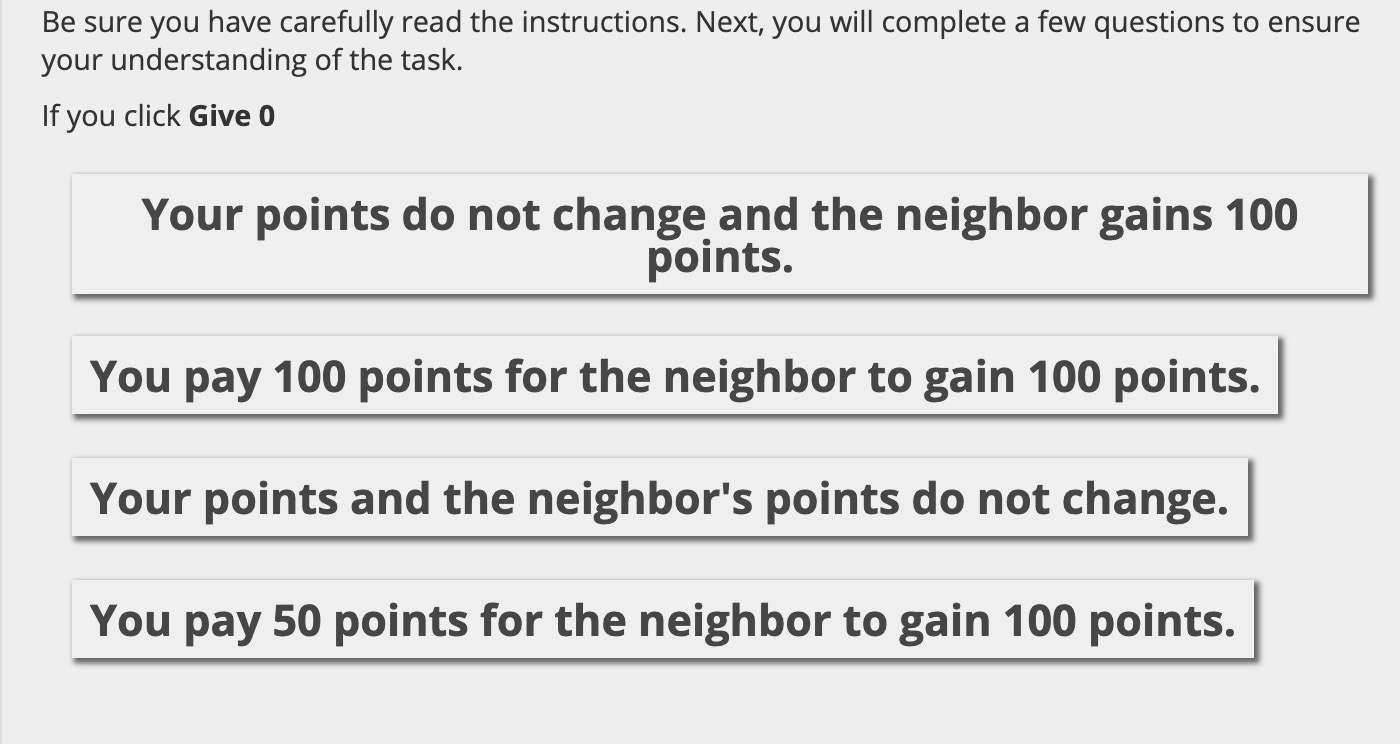


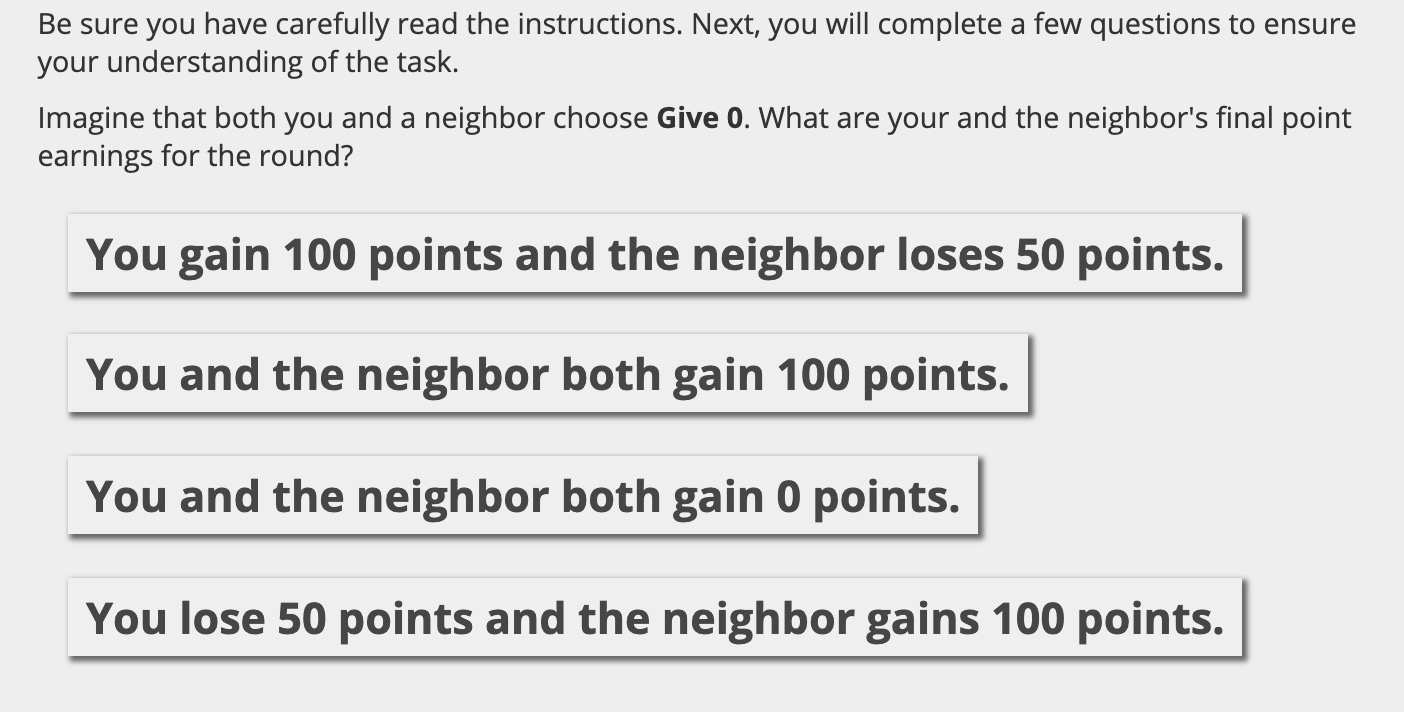


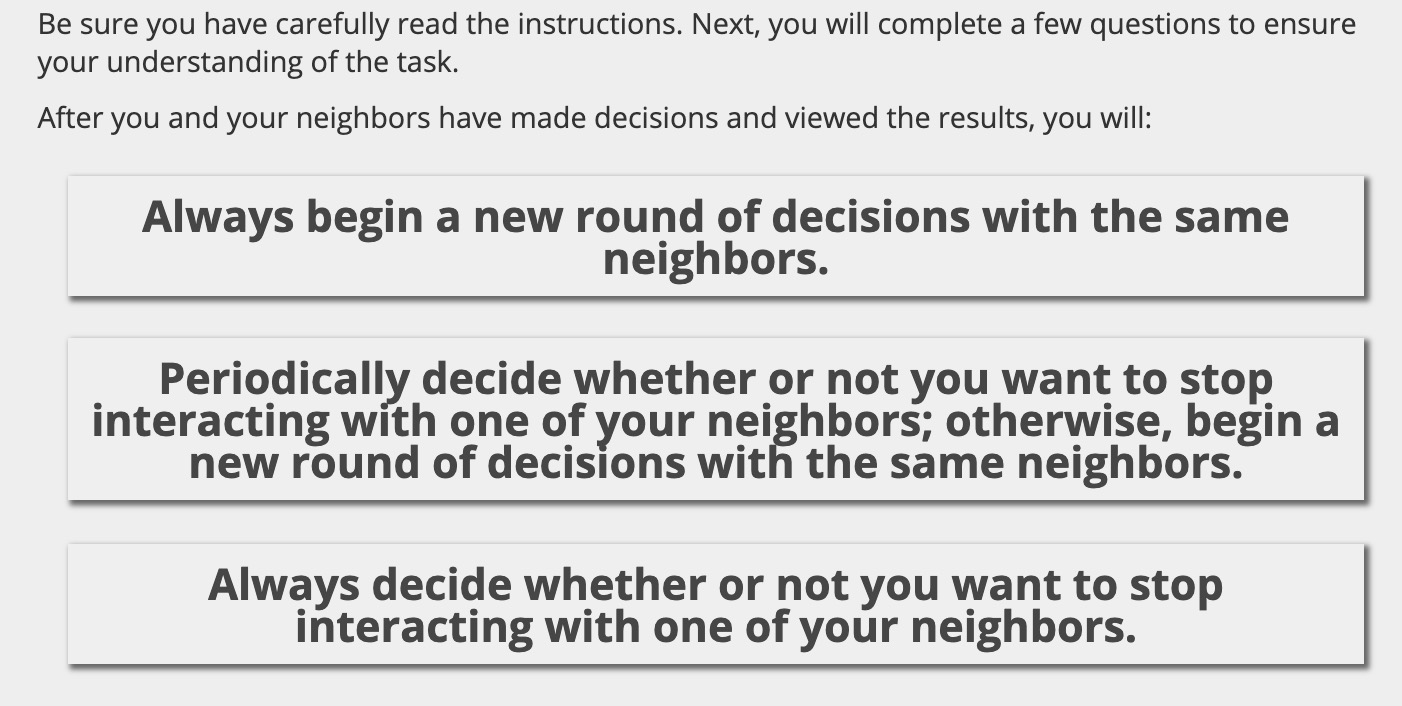


*The Iterated PD Portion*


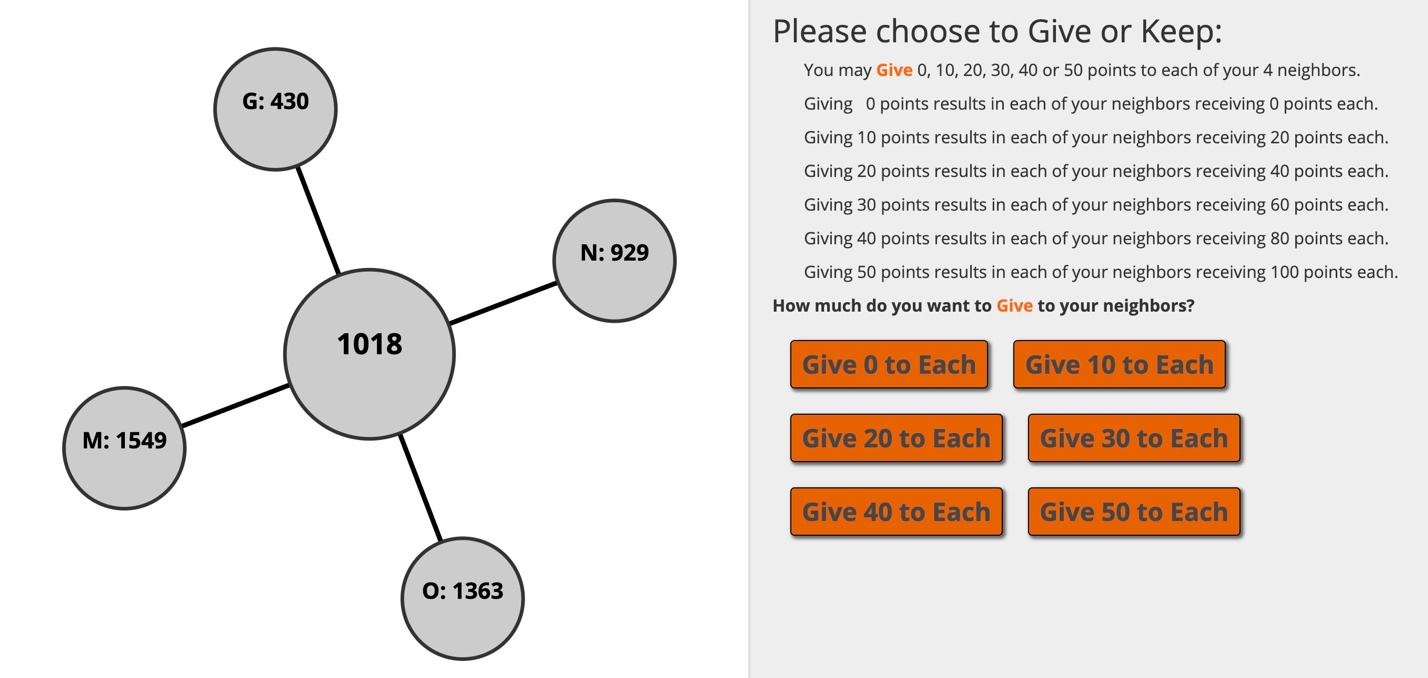


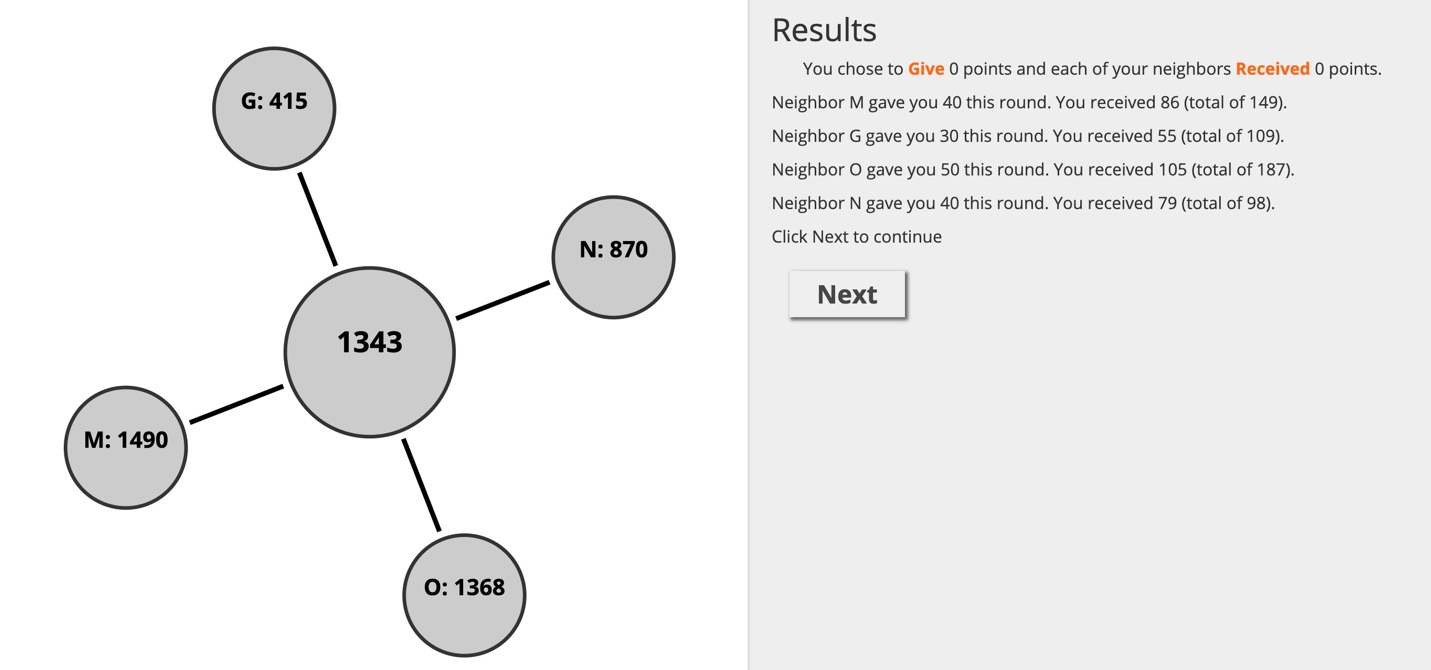


*Dynamic Network Cutting Ties*


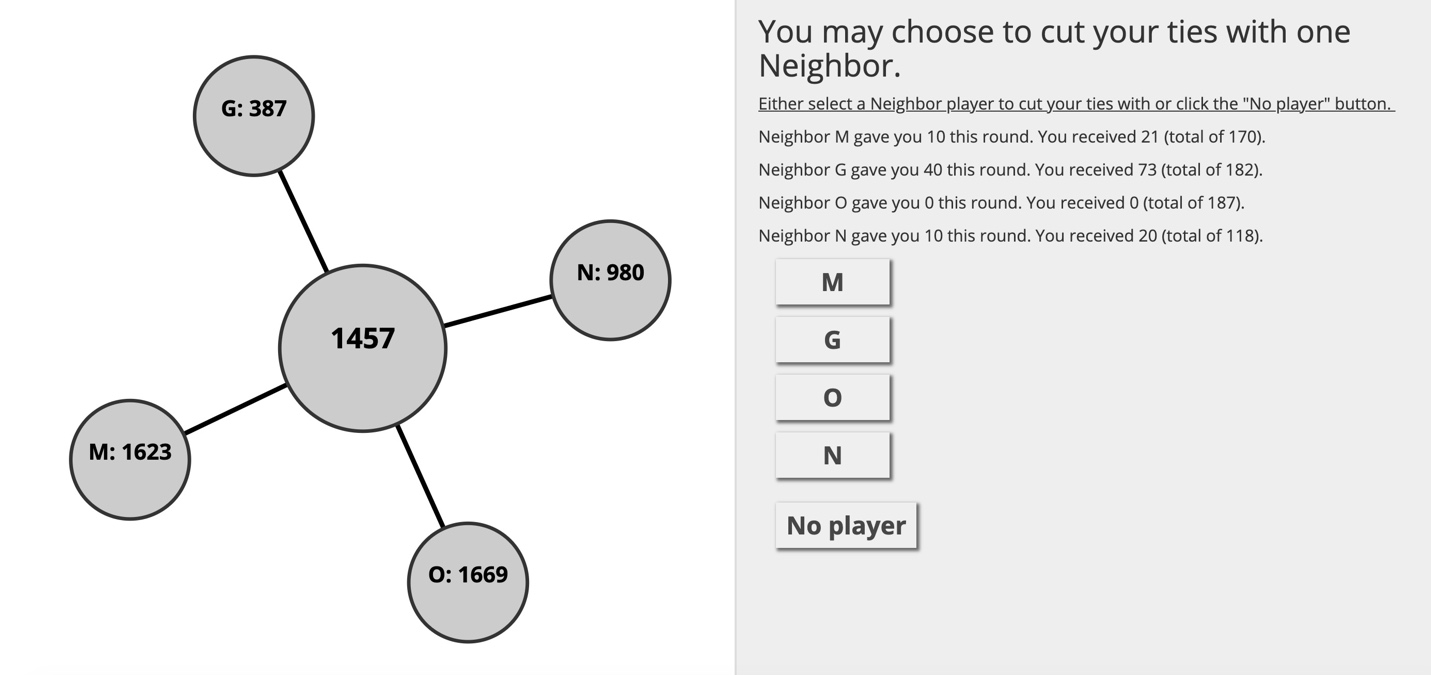


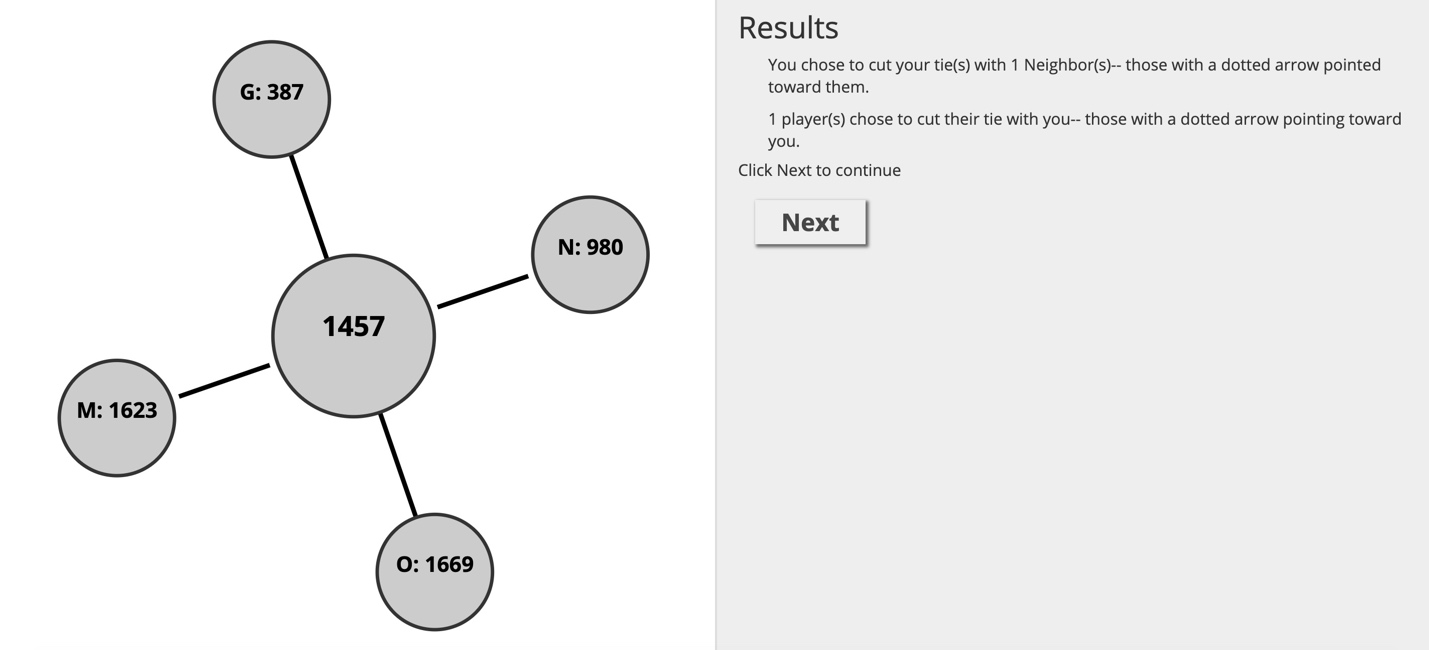


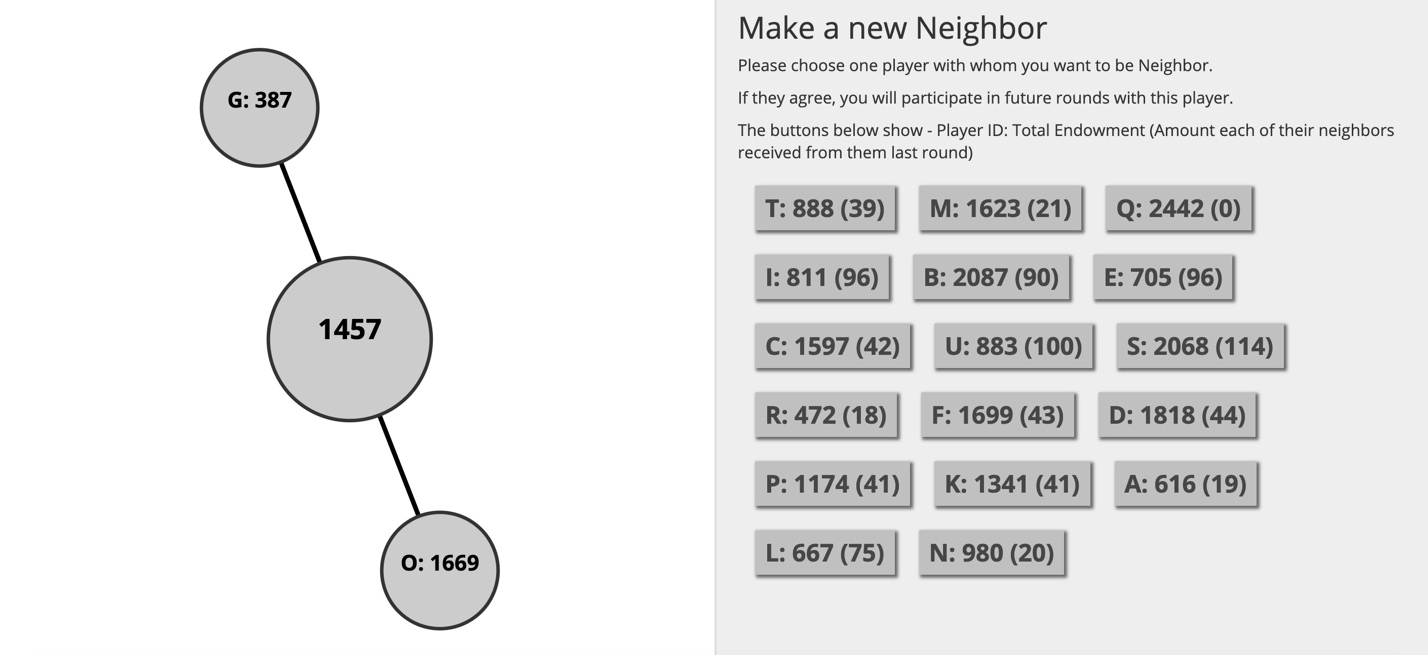


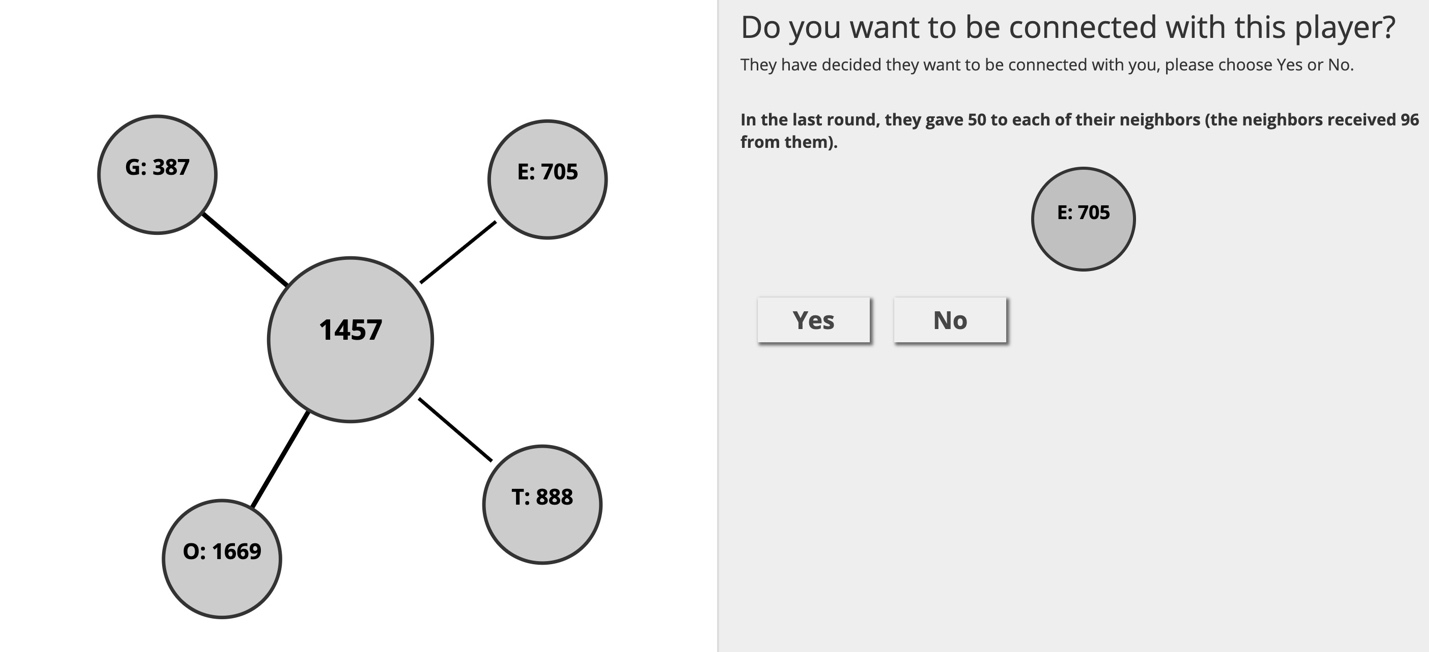


**Descriptive Statistics**

A total of 1,080 participants completed our study. Participants were 41% female. In terms of race, 79% of participants identified as white, 4% identified as Asian, 2.2% identified as Black, 12.2% identified as Hispanic/Latino, and 2.6% identified with more than one category or “other.” Figure S1 shows the distributions of participant age and education, respectively. On average, participants were 26 years old (sd = 7.8).


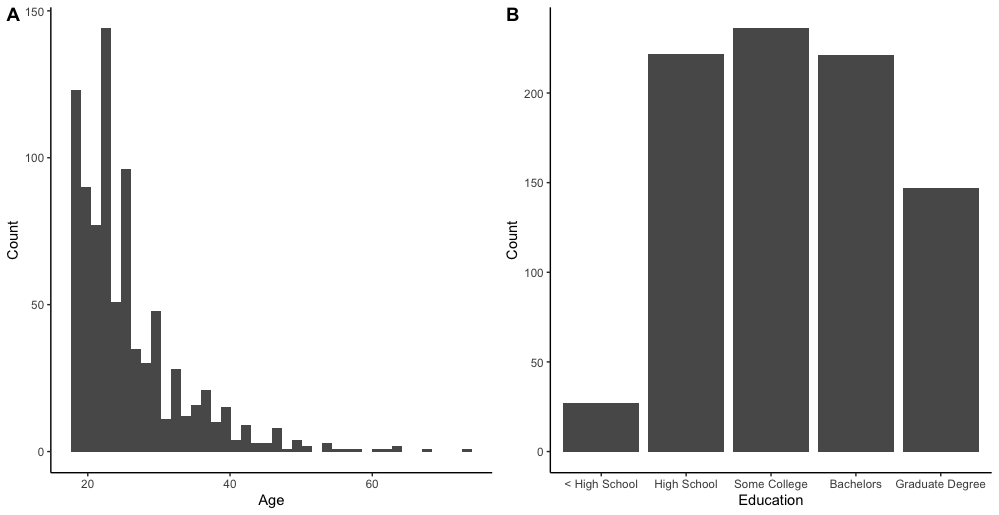


Figure S1: Descriptive statistics for age (A) and education (B).

The 1,080 participants were embedded in 40 networks. Figure S2 shows the average network size by condition over rounds of the study. The networks became smaller for a few different reasons. Any participant who was idle for 10 seconds was dropped by the program to avoid network crashes. Internet connectivity, for example, was often cited by participants who were dropped but contacted us after the study. Participants were also excluded from the game through network dynamics. Ties could be broken unilaterally and new ties were formed bilaterally. So participants could lose ties and do nothing do about it, and any ties that they proposed had to be approved. Disapproved ties were one means through which the density of the networks changed throughout the study (as were any dropped participants). Any participant with no ties at the end of a tie update phase were excluded from the network and went straight to the post-study questionnaire.

**
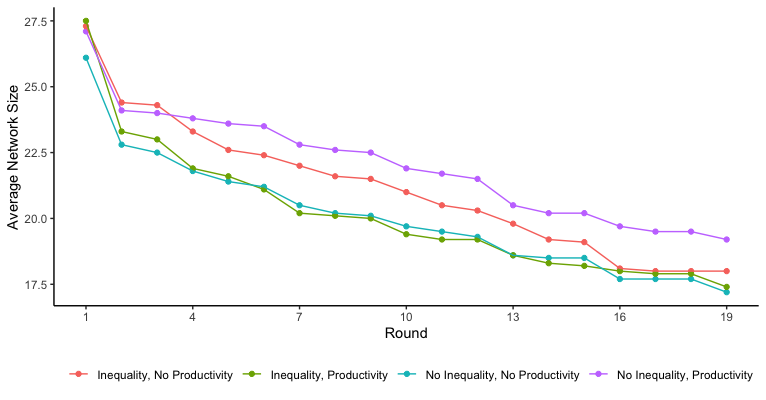
**

Figure S2: Average network size by condition and round of the experiment.

**Supplemental Data**

Figures S3-6 depict all 40 of our networks at the end of each session. Node color is proportional to giving behaviors averaged over rounds 17-19 and node size is proportional to their final endowment at the end of the game. Figure S3 illustrates the networks in the control condition. Figure S4 illustrates the networks in the endowment inequality condition without productivity. Figure S5 includes productivity without endowment inequality, and Figure S6 depicts the networks with both endowment inequality and productivity.


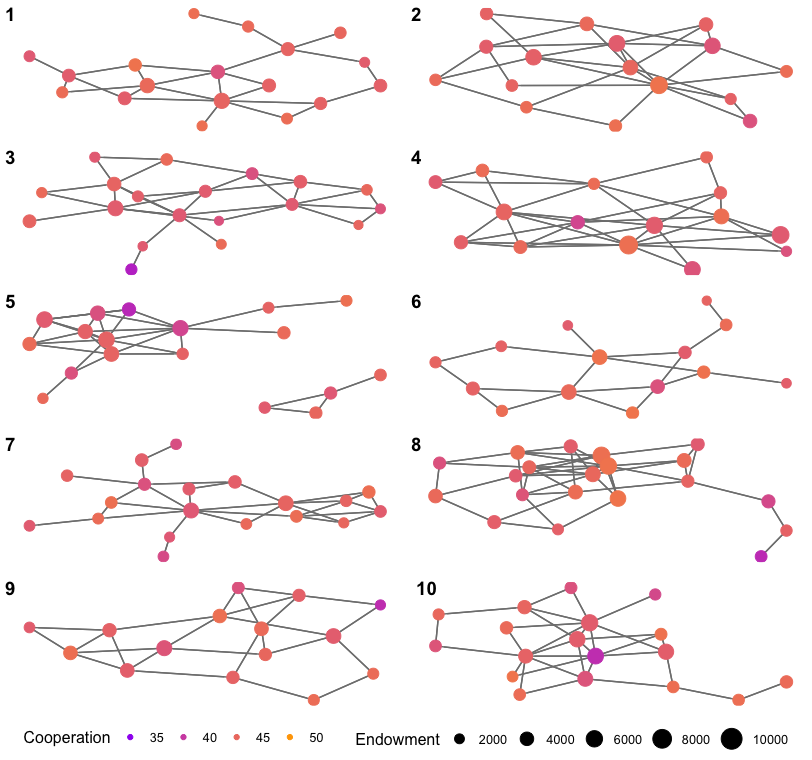


Figure S3: Networks assigned to the control condition at the end of the study.


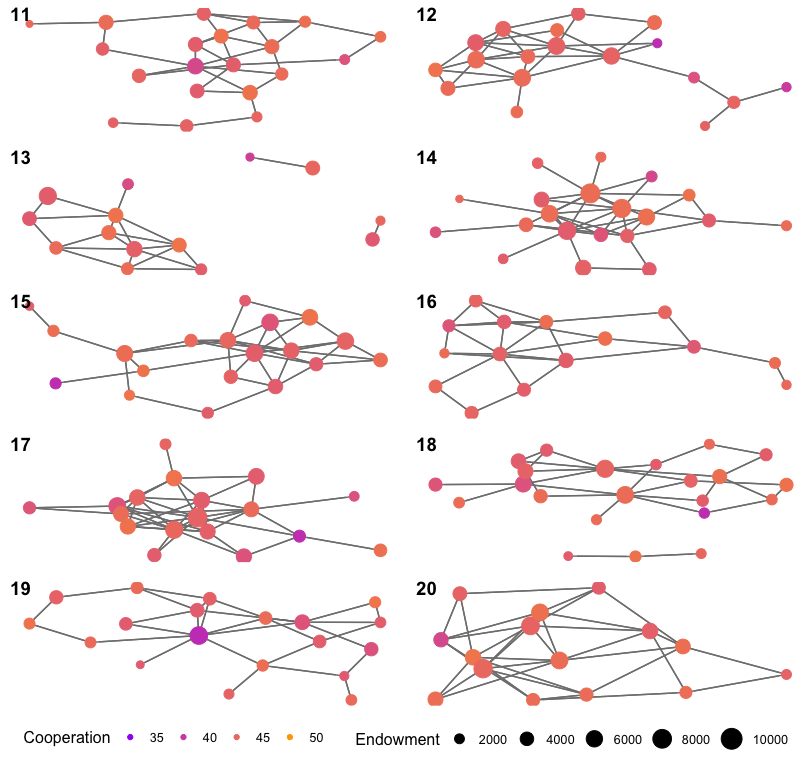


Figure S4: Networks assigned to the endowment inequality only condition at the end of the study.


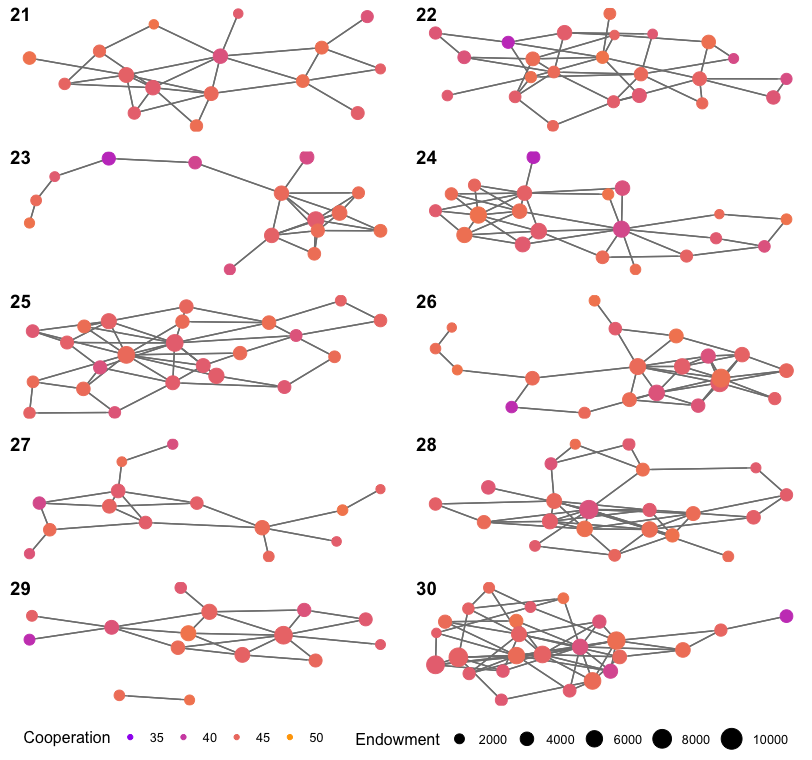


Figure S5: Networks assigned to the productivity only condition at the end of the study.


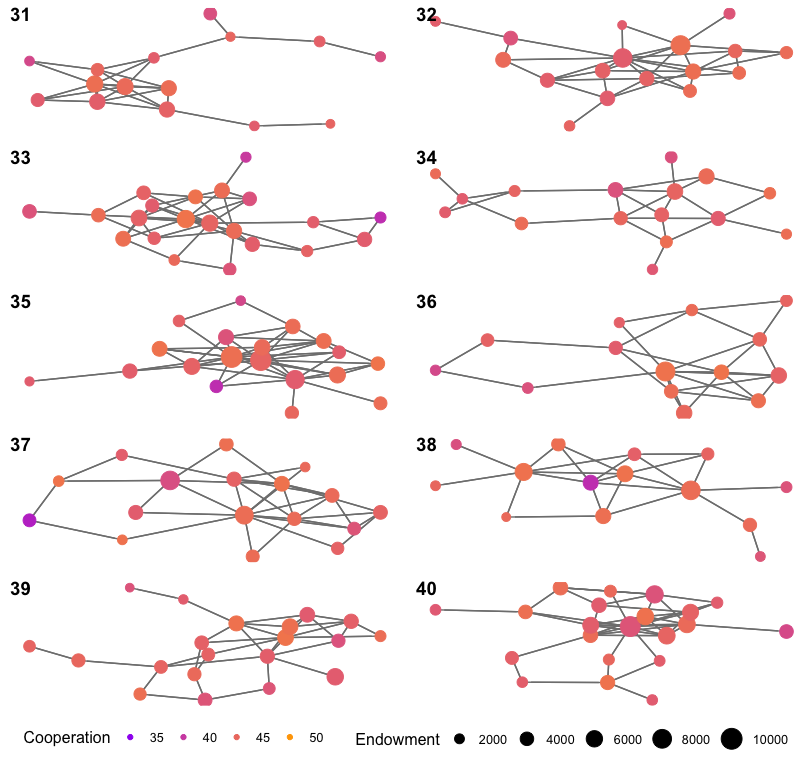


Figure S6: Networks assigned to the endowment inequality with productivity condition at the end of the study.

**Statistical Analysis**

*Cooperation*

To model cooperation, we began by looking at rates of cooperation within networks at the end of the study. Specifically, we averaged over all participants within networks over the last four rounds. Each network is therefore only 1 data point and the sample space is 40 networks. As reported in Table 1, at this level of analysis, we find that initial endowment inequality promotes cooperation, but the wealth productivity manipulation does not.

The next level of analysis is time in networks. At this level, we examine the trends that result in the patterns observed in the OLS model in the paragraph above. Here we compute average cooperation rates within network-rounds. We focus on rounds 7-19, once the trends in the data are apparent (Fig 2 A). In this case, we estimated a linear mixed model, with rounds nested in networks. Compared to a model with only main effects for round, the wealth productivity manipulation and the endowment inequality manipulation, we find that wealth productivity shapes the trajectories (i.e., interacts with experimental round; Chi-Squared = 8.83, DF = 1, *p* = .003). Further, we find that the interaction between round and wealth productivity is moderated by the endowment inequality manipulation (Chi-Squared = 34.40, DF = 4, *p* < .001). The results from this model are in Model 2, Table 1 and illustrated in Figure 4.

As noted in the main text, the patterns observed in the model above indicated that participants are attuned to the amount of inequality in their networks, particularly with respect to the effect of the wealth productivity manipulation. For each network-round, we computed the Gini coefficient corresponding to the endowment distribution. We find that respondents indeed cooperate more in the presence of wealth productivity at higher levels of inequality (interaction between wealth productivity and the Gini coefficient: b = 44.69, SE = 11.41, *p* < .001). Further, we find that this pattern does not vary with experimental round (Chi-Squared = 6.47, DF = 3, *p* = .09), meaning that regardless of when it occurs, greater inequality in the wealth productivity condition is associated with increased giving. As noted in the main text, we find that the interaction between round and the wealth productivity manipulation is moderated by the initial endowment inequality manipulation (Chi-Squared = 17.72, DF = 3, *p* < .001). Parameter estimates from this model are reported in Model 3 of Table 1.

Finally, to assess how alter endowments shape participant cooperation, we had to move down another level of analysis. Here we model individual decisions to cooperate for each person in each network. Here there is variability in the average endowment level of alters. To avoid endogeneity of endowments, we z-transformed the endowment distribution within cases and rounds. We then averaged over the participants’ partners z-scores to create a variable reflecting the average standing in the endowment distribution of the participants’ partners. We began by testing whether the effect of alter endowment varies by wealth productivity. The baseline model included main effects for experimental round, alter average endowment, an indicator for the wealth productivity condition, and an indicator for the endowment inequality condition. Compared to the baseline model, the model including the interaction between wealth productivity and alter average endowment is preferred (Chi-squared = 4.681, DF = 1, *p* = .03). We also find that the model should include an interaction between endowment inequality and alter average endowments; first we added a three-way interaction between alter endowment, wealth productivity and initial endowment inequality, but this could be constrained to only include interactions between wealth productivity and average alter endowment, and endowment inequality and average alter endowments. This is the final model in Table 1.

*Network Change*

After every third round participants had the opportunity to drop one of their alters and select a new one. The first decision we model is this binary decision to drop an alter. There were six such binary decisions nested in each participant. We modeled these repeated binary decisions using a generalized linear mixed model with a logistic link function. Parameter estimates were obtained using Maximum Likelihood estimation with a Laplacian Approximation to the log-likelihood (via lme4 in R; Bates et al. 2014). Table S1 presents parameter estimates from two model specifications. The first shows the effects of experimental manipulations on dropping an alter, and the second shows those parameter estimates with controls for time, and two parameters to adjust for direct reciprocity (*19*): how much the participant gave that round, and how much the participant received that round. We report results from Model 2 in the main text.

Table S1: Summary of a generalized linear mixed model predicting whether participants decided to drop an alter in a given round.

|  |  | Model 1 | Model 2 |
| --- | --- | --- | --- |
| Endowment Inequality |  | -.279^**^  (.106) | -.347^**^  (.112) |
| Wealth Productivity |  | .049  (.106) | .020  (.113) |
| Round |  |  | -.962^***^  (.092) |
| Round^2 |  |  | .787^***^  (.132) |
| Received from Others |  |  | .001^***^  (.000) |
| Given to Others |  |  | -.006^***^  (.001) |
| Intercept |  | -.491^***^  (.092) | 1.548^***^  (.181) |
| Variance Component |  | .077 | .082 |

*Note*: N = 4,955 participant decisions. ^*^*p* < .05, ^**^*p* < .01, ^***^*p* < .001.

Conditional on dropping an existing alter, the next decision participants faced was which one to drop. We model this decision using conditional or fixed effects logistic regression, where the conditioning is on the fact that there is only one observed “1” within a strata (Allison 2009). As with other fixed effects approaches, only within variability can be modeled because between variability is removed in the conditioning process. In our context, that implies that we can model alter giving and endowments since they will vary within participants, but that experimental condition cannot be modeled (since it is strictly between subjects). However, we can estimate an interaction effect between condition and alter endowments to trick the model into providing separate estimates for experimental conditions (Allison 2009, p. 19). The main effect of alter endowments will be the effect of alter endowment in the control condition, the main effect of being in the wealth productivity condition will be omitted by the software (due to conditioning it will have no variability), and the interaction term refers to how the main effect is modified in the experimental condition. In this way, we can assess whether preferential attachment to the wealthy is stronger in the wealth productivity condition.^^[[1]](#footnote-1)^^ The amount given is in the original metric (0-50). For comparability purposes, alter endowments were *z*-transformed (i.e., *N* ~ (0,1)) within cases and rounds.

Table S2: Summary of conditional logistic regression models predicting which alter participants dropped.

|  |  | Model 1 | Model 2 |
| --- | --- | --- | --- |
| Alter Gave Last Round |  | -.096^***^  (.003) | -.096^***^  (.003) |
| Alter Endowment |  | .138^***^  (.034) | .174^***^  (.049) |
| Alter Endowment x Wealth Productivity |  |  | -.069  (.069) |

*Note:* ^*^*p* < .05, ^**^*p* < .01, ^***^*p* < .001. N = 1,734 decisions to drop an alter.

Table S3: Summary of conditional logistic regression models predicting which alter participant’s selected.

|  |  | Model 1 | Model 2 |
| --- | --- | --- | --- |
| Alter Gave Last Round |  | .052^***^  (.003) | .052^***^  (.003) |
| Alter Endowment |  | .441^***^  (.025) | .288^***^  (.036) |
| Alter Endowment x Wealth Productivity |  |  | .295^***^  (.050) |

*Note:* ^*^*p* < .05, ^**^*p* < .01, ^***^*p* < .001. N = 1,646 decisions to select a new alter.

In light of the strong effects of wealth productivity on partner selection described in the main text, we also modeled the change in participants’ number of partners. We model change in number of partners instead of just modeling the number of partners for two reasons. First, the initial number of partners was randomly assigned. Looking at change in the number of partners removes this stochastic component. Second, change in partners is approximately normally distributed, but the number of partners is count distributed. Adjusting for three levels of nesting is far more complicated with a count distribution. We therefore regress change in number of partners (from round 4 to round 19) on the participant’s average cooperativeness, the participant’s *z*-transformed wealth at round 19, and our experimental manipulations. The model includes a random intercept for participants within networks. Table S4 presents the parameter estimates from the model. We find that those in the endowment inequality condition lose partners, more cooperative participants gain partners, and the wealthy in the wealth productivity condition gain partners (as illustrated in Figure 7).

Table S4: Summary of a linear mixed model predicting change in number of partners.

| Wealth Productivity Condition (WP) | .091  (.144) |
| --- | --- |
| Initial Endowment Inequality | -.387^**^  (.147) |
| Average Cooperativeness | .112^***^  (.008) |
| Participant Wealth (PW) | -.034  (.085) |
| WP x PW | .312^**^  (.118) |
| Intercept | -5.033^***^  (.348) |
| Variance Component | .073 |

*Note*: ^*^*p* < .05, ^**^*p* < .01, ^***^*p* < .001. N = 280 network-rounds.

*Topological Network Change:* Preferential attachment to wealthy participants should result in meso-level structural change. In particular, we expect our wealth productivity networks to increase in degree inequality as our study progressed (i.e., with time) because participants were preferentially attaching to the wealthy. Each dynamic network had 7 observations (corresponding to the initial network, and the network following each dynamic network update). For each instance of the network, we computed the inequality (Gini coefficient) in the degree distribution. Figure S7 shows the average degree inequality by experimental conditions for each round of the study. As expected, degree inequality increases over the course of our study. Table S5 presents the results from a linear mixed model predicting network degree inequality. We find that time has a squared effect on degree inequality, and that the effect of time is moderated by wealth productivity. Figure S8 shows that towards the end of the study, networks with wealth productivity indeed exhibit stronger degree inequality.


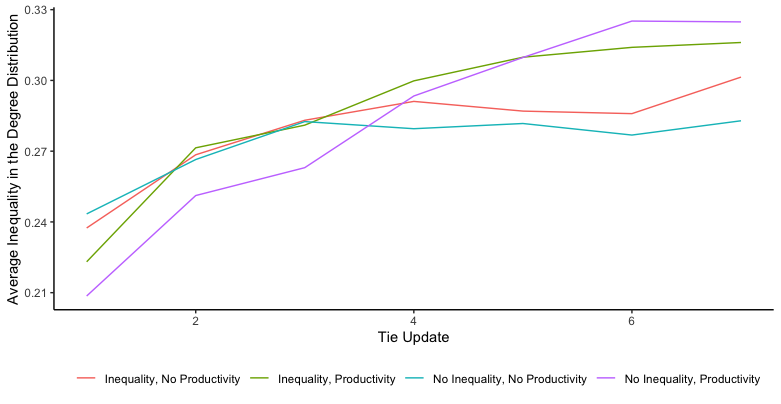


Figure S7: Average inequality (Gini) of the degree distribution (A) and core/periphery structure (B) for dynamic networks through time.

Table S5: Summary of a linear mixed model predicting inequality in network degree

| Round (R) | .007^***^  (.002) |
| --- | --- |
| Round^2 (R2) | -.001^**^  (.0005) |
| Wealth Productivity Condition (W) | -.031^*^  (.014) |
| R x W | .005^*^  (.002) |
| R2 x W | -.001  (.001) |
| Intercept | .239^***^  (.010) |
| Variance Component | .001 |

*Note*: ^*^*p* < .05, ^**^*p* < .01, ^***^*p* < .001. N = 280 network-rounds.


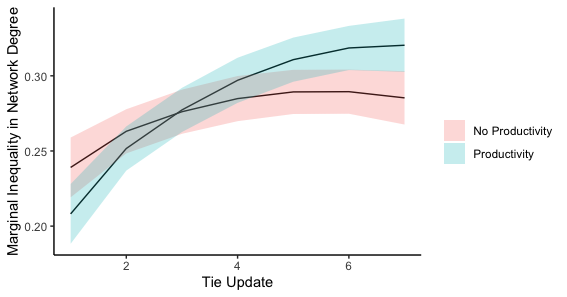


Figure S8: Marginal network degree inequality (Gini of the number of ties) as a function of time and wealth productivity.

*Network Isolation:* Participants could have dropped out of our study for a variety of reasons (e.g., loss of internet connectivity). But we also find theoretically relevant drops. 1,080 participants began our study, and 718 participants completed those conditions. Of the 362 participants who started but did not complete the study, 134 of them were excluded or isolated during network dynamic processes. That is, 134 participants began a network dynamics phase with partners, and then were excluded from the network because their partners dropped them. Table S6 shows the number of participants who were excluded/isolated and those who were not by experimental condition and tie update opportunities (see also, Fig 2B). As expected, those in the endowment inequality and wealth productivity condition appear more likely to become isolated, particularly early in the experiment. But these results do not include information about how much participants gave, which is a significant predictor of network exclusion.

Table S6: Tabulation of isolated participants by experimental condition and dynamic opportunities. Only dynamic network conditions shown.

|  | After Round | 3 | 6 | 9 | 12 | 15 | 18 |
| --- | --- | --- | --- | --- | --- | --- | --- |
| No Endowment Inequality, No Wealth Productivity | | | | | |  |  |
|  | Not Isolated | 219 | 206 | 197 | 189 | 178 | 172 |
|  | Isolated | 6 | 6 | 4 | 4 | 7 | 5 |
| Endowment Inequality, No Wealth Productivity | | | | |  |  |  |
|  | Not Isolated | 235 | 220 | 210 | 199 | 182 | 180 |
|  | Isolated | 8 | 4 | 5 | 4 | 9 | 0 |
| No Endowment Inequality, Wealth Productivity | | | | |  |  |  |
|  | Not Isolated | 238 | 227 | 219 | 205 | 197 | 192 |
|  | Isolated | 2 | 8 | 6 | 10 | 5 | 3 |
| Endowment Inequality and Wealth Productivity | | | | |  |  |  |
|  | Not Isolated | 221 | 200 | 194 | 187 | 180 | 174 |
|  | Isolated | 9 | 11 | 6 | 5 | 2 | 5 |

As illustrated in Fig S9, those who were excluded from the endowment inequality and wealth productivity condition gave more than those in other conditions (purple line). That is, participants in the endowment inequality and wealth productivity condition were more likely to be excluded from the network, despite giving more than isolates in other conditions.


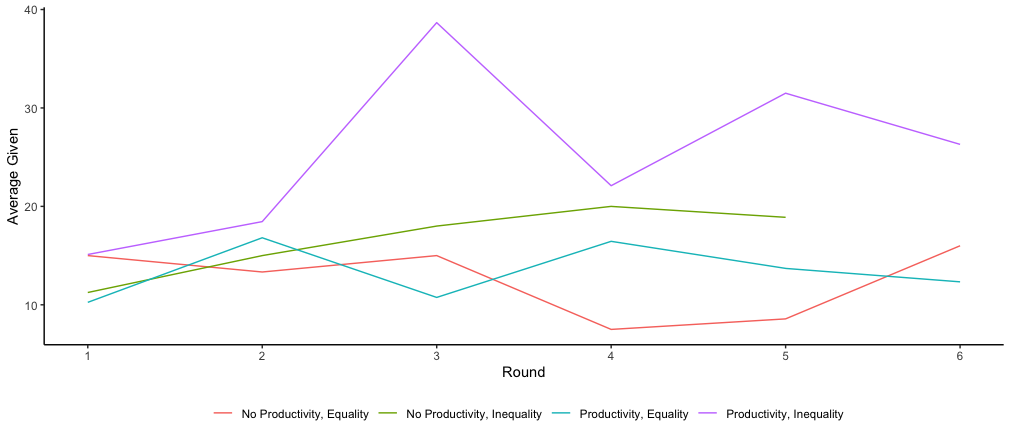


Figure S9: Average amount given for those who were isolated/excluded from the dynamic networks by experimental condition and dynamic network update opportunities.

We conducted a statistical analysis of the amount given by those who were excluded from the study. Dynamic network update opportunities were not predictive at all (F_(1,5)_ = .67, *p* = .65), and so were excluded from our final model. Isolated participants in the endowment inequality and wealth productivity condition gave more than isolated participants in the control (b = 9.08, *p* = .012) and only wealth productivity condition (b = 8.64, *p* = .016). Isolated participants in the endowment inequality and wealth productivity condition also gave more than isolated participants in the endowment inequality only condition, but not significantly so (b = 5.246, *p* = .152).

In order to formally determine condition-level effects on participants becoming isolated, we estimated Cox proportional hazards models using R’s Survival package (Therneau 2021). Table S7 presents the results from four hazard models. Model 1 only includes experimental conditions. None of the coefficients are significant. Model 2 includes two time-varying covariates – (1) how much the participant gave on the preceding round (which was provided to participants on their screens), and (2) the participant’s endowment at the end of the preceding round (also shown to participants during network updates). Model 2 shows that participants were less likely to be isolated from the network the more they gave/cooperated, and the larger their endowment. Importantly, the control variables result in the effect of being in the endowment inequality and wealth productivity condition becoming significant. Model 3 and 4 include only one control at a time to illustrate which of the controls is suppressing the effect of experimental condition. Once giving is controlled (Model 4), participants were more likely to be excluded from the endowment inequality and wealth productivity networks.

Table S7: Summary of Cox proportional hazards models predicting isolation from our dynamic networks.

|  | Model 1 | Model 2 | Model 3 | Model 4 |
| --- | --- | --- | --- | --- |
| Endowment Inequality, No Wealth Productivity^1^ | -.118  (.235) | .375  (.401) | -.121  (.275) | .428  (.368) |
| No Endowment Inequality, Wealth Productivity^1^ | -.035  (.269) | .119  (.390) | -.006  (.272) | .080  (.378) |
| Endowment Inequality, Wealth Productivity^1^ | .175  (.184) | .942^**^  (.352) | .197  (.197) | .921^**^  (.329) |
| Endowment^2^ |  | -.001^***^  (.000) | -.001^***^  (.000) |  |
| Cooperation^2^ |  | -.093^***^  (.004) |  | -.094^***^  (.004) |

*Note*: ^*^*p* < .05, ^**^*p* < .01, ^***^*p* < .001. N = 4,955 participant-rounds at risk. ^1^Reference category is No Endowment Inequality, No Wealth Productivity. ^2^Time-varying covariate. Robust standard errors are reported (clustered on the network).

*Inequality*

We observe a restricted range of inequality in our networks. This led us to question how much inequality is possible in the social systems we studied. While inequality (i.e., the Gini coefficient) theoretically ranges from 0 to 1, at the end of the study the observed range is .12 to .26. The effects of our manipulations on inequality are therefore unclear: if the system cannot produce much inequality, perhaps our effects are large in magnitude, but if the system can produce a lot of inequality, but we do not observe it, then we know our effects are relatively negligible. In order to have a sense of how much inequality is possible, we conducted a numerical simulation. We simulated a random network with 20-28 nodes and a density of .167 (the same as in our experiment). We then randomly allocated giving decisions to the nodes (from 0 to 50 in 10-point increments) and computed the updated endowments. We repeated the process of selecting amounts to give and updating the endowments five additional times (also in line with our experimental data). In the numerical simulation, we varied the initial endowment of nodes. In half the simulation, nodes all received endowments of 1,000 (same as our control condition). In the other half, the initial endowment distribution had a mean between 950 and 1,050, had a gini coefficient between .25 and .35, and all nodes had a positive endowment (similar to our endowment inequality condition).

Figure S10 shows the distribution of observed gini coefficients after the agents made their six cooperation decisions. Under conditions of initial equality, we observe an average gini coefficient of .12 with a .02 standard deviation, and 95% of the distribution is between .084 and .158. In cases with endowment equality in our data, averaging over rounds 16-19, we observe an average gini coefficient of .15 in the no wealth productivity condition and an average gini coefficient of .17 in the wealth productivity condition. The difference between conditions corresponds to a standard deviation in the simulated distribution, which is a sizable effect. Under conditions of initial inequality, in the simulation we observe an average gini coefficient of .21 with a .03 standard deviation, and 95% of the distribution is between .16 and .26. In cases with endowment inequality we observe an average gini coefficient of .19 in the no wealth productivity condition and an average gini coefficient of .21 in the wealth productivity condition. Here the difference between conditions corresponds to ⅔ of a standard deviation, another relatively large effect.


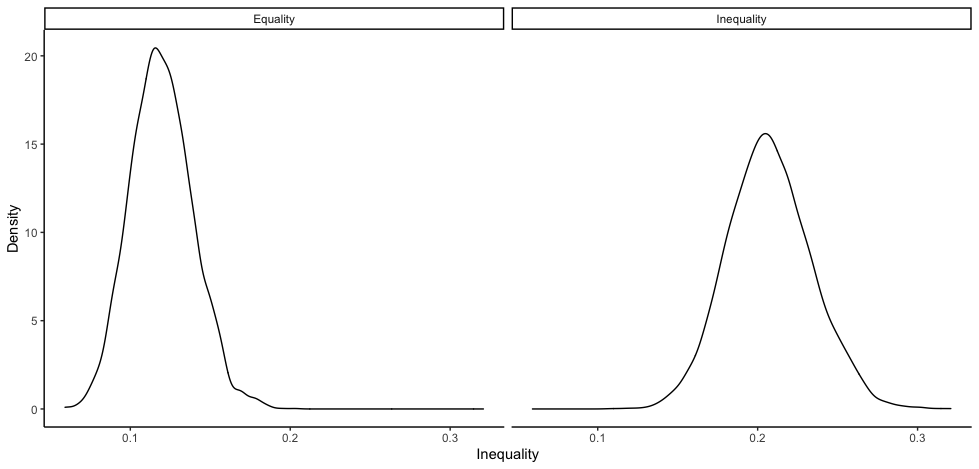


Figure S10: Density plots of observed inequality (Gini coefficients) in our numerical simulation.

On the one hand, the fact that the agents in the simulation above behaved randomly implies that there would be more inequality than in our behavioral data, since participants were more likely than not to cooperate. On the other hand, however, the distribution of behaviors should be structured by the network, rather than at random. This implies that the estimates of inequality are conservative because there are no network effects (ties and behavior are random). To address both of these concerns, we ran a second numerical simulation. Instead of using random networks, we used clustered networks (Watts 1999, with *p* = .2). Each node was assigned to a community (Girvan and Newman 2002). For each community, we sampled one amount to give and every node in that community gave that amount, repeatedly. This builds strong network effects into the simulation in that each node within a community gives at the same rate. Over time this will increase between group inequality. Figure S11 presents density plots of observed inequality after 6 rounds of cooperation and endowment updates. We observe slightly more inequality than above, but not much more. Under conditions of strong network effects, but initial equality, we observe an average gini coefficient of .13 with a .03 standard deviation, and 95% of the distribution is between .07 and .21. Simulated networks with initial endowment inequality results in an average gini coefficient of .19 with a .04 standard deviation, and 95% of the distribution is between .13 and .27. Using 95% of the distributions of inequality as a guide, we find that the social systems we study produce a viable range of Gini coefficients between .07 and .27.


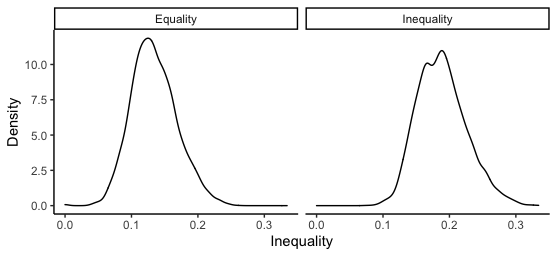


Figure S11: Density plots of observed inequality (Gini coefficients) in our second numerical simulation.

In terms of modeling inequality, as above we begin modeling inequality by computing the Gini coefficient. We first computed the Gini coefficient for each network-round. We then averaged over the last 4 rounds to smooth any irregularities in the final round. At this network level of analysis (N=40), we find that initial endowment inequality is strongly related to inequality at the end of the study (Table S8, Model 1: b = .044, se = .009, *p* < .001). We also find that networks with the wealth productivity manipulation have increased inequality at the end of the study (b = .016, se = .009, *p* = .08). Importantly, we point out that our measure of inequality is on the adjusted endowment distribution that corrects for the role of the wealth productivity manipulations on participant endowments.

We also modeled trends in inequality through time. We find that the effect of round is moderated by wealth productivity (b = .002, se = .000, *p* < .001), and that this interaction between round and wealth productivity is moderated by the endowment inequality manipulation (Chi-Squared = 167.36, DF = 3, *p* < .001). Parameter estimates from this model with the three-way interaction are presented in Model 2 of Table S8. Figure 9 (main text) shows marginal inequality from the model. In both cases we see that wealth productivity increases inequality through time.

Table S8: Summary of regression models predicting network-level inequality

|  |  | Model 1 | Model 2 |
| --- | --- | --- | --- |
| Wealth Productivity Treatment (W) |  | .016^a^  (.009) | -.017  (.011) |
| Initial Endowment Inequality (I) |  | .044^***^  (.009) | .103^***^  (.011) |
| Round (R) |  |  | .003^***^  (.000) |
| W $\times$ I |  |  | .014  (.016) |
| W $\times$ R |  |  | .002^***^  (.000) |
| I $\times$ R |  |  | -.003^***^  (.000) |
| W $\times$ I $\times$ R |  |  | -.001^*^  (.000) |
| Intercept |  | .150^***^  (.008) | .090  (.008) |
| Level 2 Variance Component |  |  | .001 |

*Note: ^a^p* = .08, ^*^*p* < .05, ^**^*p* < .01, ^***^*p* < .001. Model 1, N = 40 networks. Model 2, N = 520 network-rounds.

References

McKnight, Mark E., and Nicholas A. Christakis. *Breadboard.* Computer software.

*Breadboard: Software for Online Social Experiments.* Vers. 2. Yale University, 1 May

2016. Web.

Bates, D., Mächler, M., Bolker, B. & Walker, S. Fitting linear mixed-effects models using

lme4. *arXiv preprint arXiv:1406.5823* (2014).

Allison, P. D. *Fixed effects regression models*. (SAGE publications, 2009).

Therneau T. *A Package for Survival Analysis in R*. R package version 3.2-13 (2021),

<https://CRAN.R-project.org/package=survival>.

Watts, D. J. Networks, dynamics, and the small-world phenomenon. *American Journal of*

*sociology* **105**, 493–527 (1999).

Girvan, M. & Newman, M. E. Community structure in social and biological networks.

*Proceedings of the national academy of sciences* **99**, 7821–7826 (2002).

1. While this approach enables us to test our hypotheses about wealth productivity and network dynamics, it ignores that there are repeated decisions nested in participants and that participants are then nested in networks. Robust inference is unavailable in the context of the interactive models required. [↑](#footnote-ref-1)
